# Network medicine analysis for dissecting the therapeutic mechanism of consensus TCM formulae in treating hepatocellular carcinoma with different TCM syndromes

**DOI:** 10.3389/fendo.2024.1373054

**Published:** 2024-08-15

**Authors:** Kai Gao, WanChen Cao, ZiHao He, Liu Liu, JinCheng Guo, Lei Dong, Jini Song, Yang Wu, Yi Zhao

**Affiliations:** ^1^ School of Traditional Chinese Medicine, Beijing University of Chinese Medicine, Chaoyang District, Beijing, China; ^2^ New York Institute of Technology College of Osteopathic Medicine, Arkansas State University, Jonesboro, AR, United States; ^3^ The Research Center for Ubiquitous Computing Systems (CUbiCS), Institute of Computing Technology, Chinese Academy of Sciences, Beijing, China

**Keywords:** traditional Chinese medicine, TCM syndrome type, natural compounds, network medicine, hepatocellular carcinoma, consensus guideline

## Abstract

**Introduction:**

Hepatocellular carcinoma (HCC) is a major cause of cancer-related mortality worldwide. Traditional Chinese Medicine (TCM) is widely utilized as an adjunct therapy, improving patient survival and quality of life. TCM categorizes HCC into five distinct syndromes, each treated with specific herbal formulae. However, the molecular mechanisms underlying these treatments remain unclear.

**Methods:**

We employed a network medicine approach to explore the therapeutic mechanisms of TCM in HCC. By constructing a protein-protein interaction (PPI) network, we integrated genes associated with TCM syndromes and their corresponding herbal formulae. This allowed for a quantitative analysis of the topological and functional relationships between TCM syndromes, HCC, and the specific formulae used for treatment.

**Results:**

Our findings revealed that genes related to the five TCM syndromes were closely associated with HCC-related genes within the PPI network. The gene sets corresponding to the five TCM formulae exhibited significant proximity to HCC and its related syndromes, suggesting the efficacy of TCM syndrome differentiation and treatment. Additionally, through a random walk algorithm applied to a heterogeneous network, we prioritized active herbal ingredients, with results confirmed by literature.

**Discussion:**

The identification of these key compounds underscores the potential of network medicine to unravel the complex pharmacological actions of TCM. This study provides a molecular basis for TCM’s therapeutic strategies in HCC and highlights specific herbal ingredients as potential leads for drug development and precision medicine.

## 1 Introduction

According to the 2020 global cancer statistics report, primary liver cancer is the sixth most diagnosed cancer and the third leading cause of cancer death worldwide (1). Hepatocellular carcinoma (HCC) is the most common type of primary liver cancer, accounting for 75% to 85% of cases. China remains one of the high-risk HCC countries due to the high incidence of chronic hepatitis B and high exposure to aflatoxins. The treatment of HCC, such as surgery, radiotherapy, and chemotherapy, are usually applied in clinics based on stages (2). Among them, pharmacological intervention is often used in patients with advanced liver cancer to reduce tumor burden and prolong survival. The first- and second-line drugs, such as sorafenib and nivolumab, show relief of symptoms and prolonged survival time, but there are many adverse reactions. And the overall prognosis is still poor (3–5). In China, many patients are recommended the combinational therapies of both modern medicine and traditional Chinese medicine (TCM), aiming to improve tumor-related symptoms, enhance the body’s immunity, reduce adverse reactions from radiotherapy and chemotherapy, and improve patients’ quality of life (6, 7).

**Figure 1:**
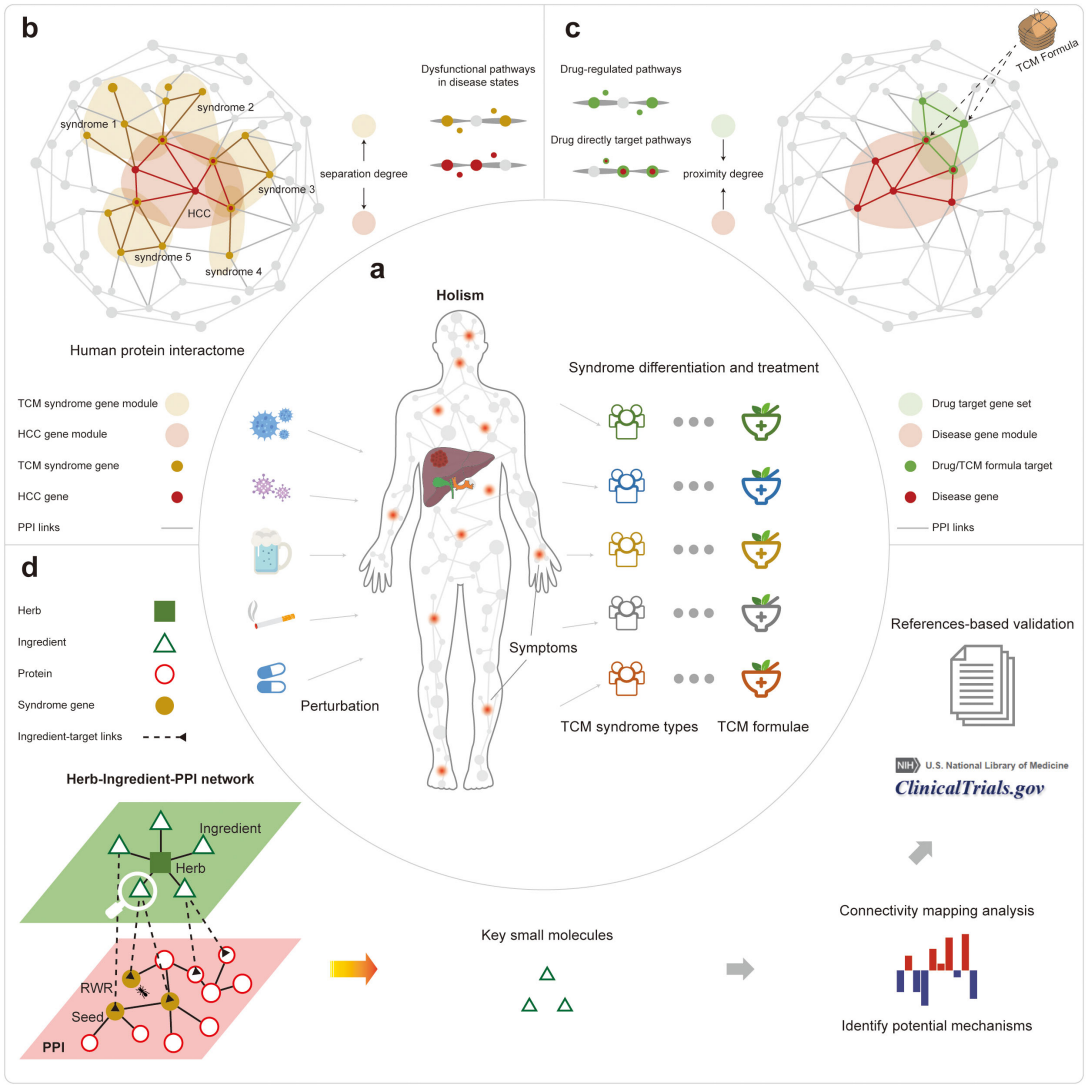
The overall workflow of this study. **(A)** The illustration of the holistic TCM theory about syndrome differentiation and the corresponding treatment. **(B)** The comparison of different TCM syndromes to the HCC diseases based on their related protein sets in the PPI network. **(C)** The comparison of different TCM formulae to the HCC disease based on their related protein sets in the PPI network. **(D)** The results of prioritization for active small molecules from different formulae treating different HCC syndrome types.

TCM has been used to treat chronic liver disease and malignant tumors for thousands of years (8). Although modern medicine has developed rapidly, TCM is still an indispensable complementary therapy in clinical practice. A recent clinical study has shown that the use of TCM as adjuvant therapy could prolong the median survival time (37 months with TCM *vs.* 9.23 months without TCM) and improve the five-years overall survival (26.4% with TCM *vs.* 10.1% without TCM) among HCC patients (9). This approach could benefit from the detailed syndrome differentiation of TCM and its holistic way for treating each of them. Because TCM considers the human body as well as the external environment in an integrative way, and regards different symptoms of the human body as the changes of the whole system in different directions. If a group of symptoms frequently appears together in patients, this specific symptom cluster will stand for a particular subtype of disease that can be stably differentiated. These symptom clusters have already been carefully summarized across thousands of years of TCM clinical observations, which were specially called TCM syndromes. For example, the disease HCC is usually classified into 5 different syndrome types according to the specific principle of TCM to perform syndrome differentiation. And then, the corresponding TCM treatment modalities were decided according to the differentiation of TCM syndromes (**Figure 1A**). In most cases, the TCM treatments were in the form of formulae, which were combinations of herbs designed for alleviating different symptoms in the TCM syndrome. For these 5 consensus syndrome types of HCC, experts recommended 5 different formulae respectively. And this recommendation is published in the 2019 edition of the “*Guidelines for the Diagnosis and Treatment of Hepatocellular Carcinoma*”, issued by the national health commission of the Chinese government (10, 11). The guideline for the HCC syndromes and formulae verified by authorized experts is formed from long-term clinical practice experience with TCM. But the underlying mechanism for this syndrome-formulae relationship is still unclear.

One efficient way for dissecting the underlying mechanism for TCM is based on network medicine, which analyzed the drug actions in the network biology context for tackling complex diseases (12). Based on large-scale biomolecular networks, the biological regulation processes underlying the treatment of Chinese herbs, as well as their combinations as formulae can be untangled in a multi-component, multi-target, and multi-pathway way. Further, the active components and the key targets of herbs and formulae can be accurately predicted (13–15). Currently, most network medicine analysis is based on the protein-protein interactions (PPI) networks, as proteins are the major players in biological functions (16). Based on two assumptions, the potential mechanism of different TCM formulae acting on HCC with distinct syndrome types can be analyzed through their related PPI network modules, and characterized by their specific scenarios about network topologies and protein functions. The first assumption is that disease- or TCM syndrome-related proteins would be functionally correlated, thus distributed in closely connected sub-networks, or modules, in the entire human PPI network. The second assumption is that effective drugs against diseases would target proteins that were located nearby disease- or TCM syndrome-related network modules.

In this work, we explored the mechanism of TCM treatments for different syndrome types in HCC based on the information from the consensus guideline. We used network topological analysis and functional enrichment analysis of the disease-, TCM syndrome-, and the formulae-related PPI sub-networks. Firstly, we showed that the five TCM syndrome types-related genes are topologically and functionally close to the HCC disease-related genes in the PPI network (**Figure 1B**). Secondly, we quantified the proximity between the targeted proteins of the five formulae and that of different HCC syndrome types in the PPI networks, giving objective and quantitative measures about the efficiency of TCM syndrome differentiations as well as their corresponding treatment modalities (**Figure 1C**). Thirdly, we interpreted the quantitative measures of the proximity in a functional view of gene sets. Then we ranked the importance of active herbal ingredients that constitute TCM herbs by the random walk algorithm through a newly assembled heterogeneous network. The top-ranked results can be further confirmed by literature, demonstrating the power of our analysis for decoding the material basis for treating HCC (**Figure 1D**).

## 2 Materials and methods

### 2.1 Data source about the disease and TCM syndrome-related genes

We collected the disease-related gene set for HCC from the DisGeNet (http://www.disgenet.org/) and the GeneCards (https://www.genecards.org/) database. Only genes that appeared in both datasets were retained in this study to ensure data reliability. For five TCM syndromes of HCC and other syndromes, their gene sets were collected from the SymMap (http://www.symmap.org/) database, which has already linked TCM symptoms to gene targets through symptom mapping followed by statistical verification (17).We extracted statistically significant relationship data from the database, i.e. screening with a threshold of *P-value* < 0.05. The datasets of disease/syndrome -related genes/proteins are provided in **Supplementary Data S1**.

### 2.2 Data source about disease and TCM syndrome-related drugs/formulae

We collected the disease-related small molecule drugs for HCC, liver disease, and cancer based on the clinical practice guidelines from the European Society for Medical Oncology (ESMO) (18), the European Association for the Study of the Liver (EASL) (19), and the DrugBank database. For five TCM syndromes of HCC, their associated treatments as TCM formulae were collected from the 2019 version of Chinese guidelines for the diagnosis and treatment of HCC (10). The datasets of disease/syndrome-related drugs/formulae are provided in **Supplementary Data S2**.

### 2.3 Data source about drug/formula targets

We collected the targets for small molecule drugs of HCC, liver disease, and cancer from the DrugBank database (https://go.drugbank.com/). For TCM formulae, we curated their potential targets through their herb/ingredient constituents based on the curated targets in our previously constructed HERB database (20). We also quantitatively estimated the drug-likeness metric for these herbal ingredients using the *RDKit* python package (version 2023.03.1) and screened for potential active compounds in the medicinal herbs. The process of determining the quantitative estimation of drug-likeness (QED) threshold is shown in **Supplementary Appendix S1** (**Supplementary Figure S1**). The datasets of drug/formulae targets are provided in **Supplementary Data S3**.

### 2.4 The integration of a comprehensive human PPI network

We integrated a comprehensive human PPI network from six different sources of experimentally verified data. It includes: 1) HuRI: Known for its binary protein interactions that are typically direct, this bioinformatics resource maps the biophysical human protein interactome using yeast two-hybrid systems, further validated through orthogonal assays, ensuring a high degree of confidence in the interactions (21); 2) BioPlex: With many interactions involving large protein assemblies detected by affinity purification mass spectrometry (22); 3) Interactome3D: This database integrates interaction data from primary pathway repositories, compiling experimentally verified binding events (23); 4) Insider: As a tool that bridges genomic variation information with the structural protein-protein interactome, Insider provides interactions that have been determined experimentally (24); 5) PhosphoSitePlus: Encompassing a dataset of 12,180 high-quality, manually curated kinase-substrate interactions (25); 6) InnateDB: Beyond its 18,000 manually managed interactions, InnateDB also includes 178,000 experimentally verified imported interactions and 3,000 pathways (26).

Then we uniformly mapped all proteins in the network to Entrez gene IDs with official gene symbols based on the R package ‘*org.Hs.eg.db*’ (version 3.18.0) (27). Next, we calculated the largest connected component of the PPI network using the python package ‘*NetworkX*’ (version 3.1) (28). As a result, we obtained a large human interactome including 311,499 unique PPI interactions linking 17,345 proteins/genes. We mapped all drug targets in the *DrugBank* database (29) to this network and found that 94.24% of the drug targets were mappable, indicating that our network is capable of analyzing drugs based on their targets. The PPI network used in this study is provided in **Supplementary Data S4**.

### 2.5 Network-based separation analysis between HCC disease and TCM syndromes

**Figure 2:**
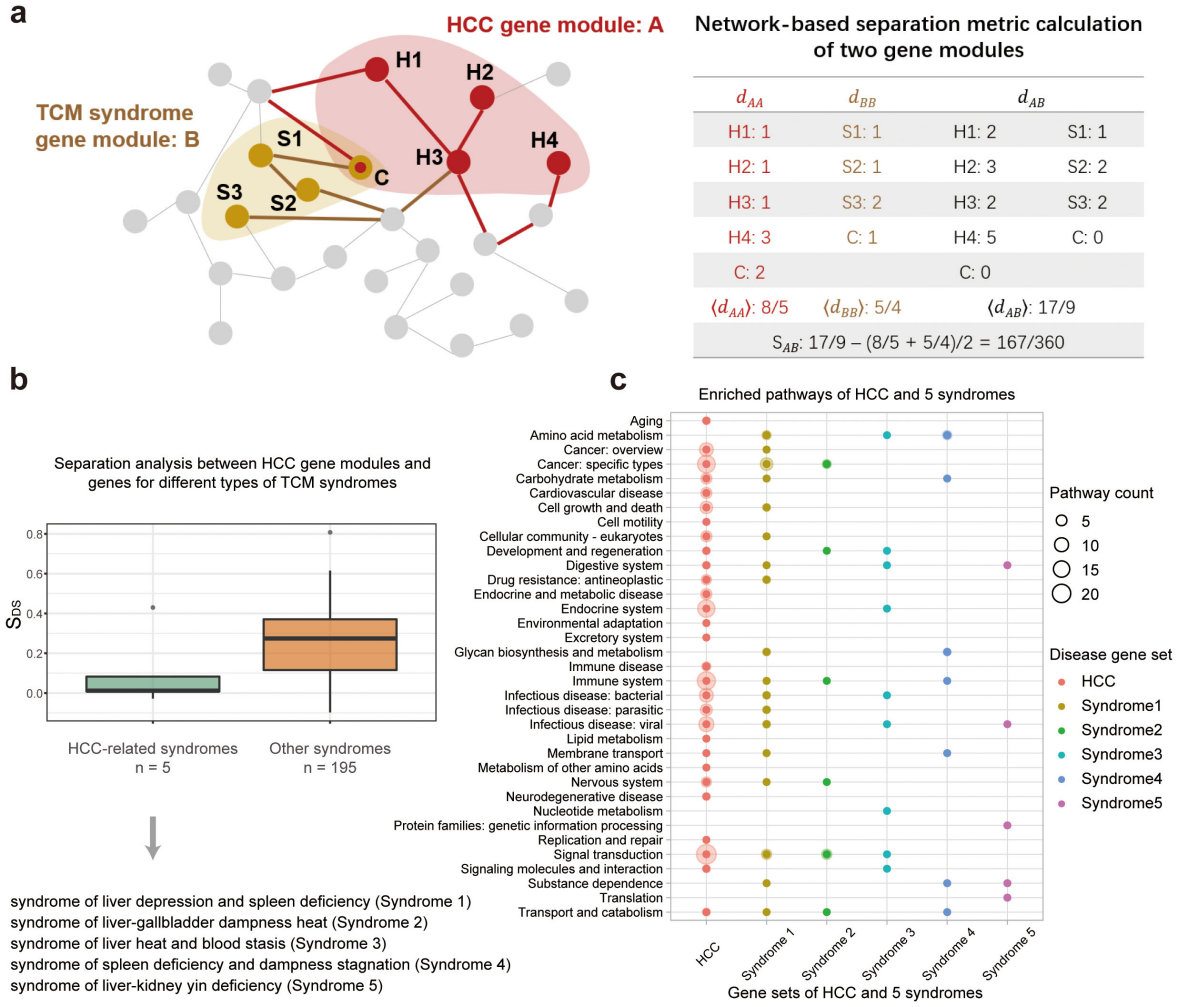
Comparison of gene sets associated with HCC disease and its 5 related TCM syndromes. **(A)** Illustration of the network-based separation analysis between HCC-related gene module and TCM syndrome-related gene modules. **(B)** Distribution of the separation measures, *S_DS_*, between genes of HCC and that of TCM syndromes. Two boxes demonstrate two types of comparison. The first box in green measures the network overlap between genes of HCC and that of the HCC-related TCM syndromes. And the second box in orange measures network overlaps between genes of HCC and that of 195 other syndromes. **(C)** Enriched KEGG pathways for genes related to HCC disease and its associated HCC syndromes. These pathways were clustered into 35 classes according to KEGG orthology.


Similar diseases exhibit similar patterns in both phenotypes and molecular mechanisms (30, 31). In the disease/TCM syndrome-related network, it was expected that diseases with similar clusters of symptoms will be closely related to adjacent network modules, and vice versa. To verify it, we quantified the distances between the HCC disease (D) and five TCM syndrome (S) related subnetworks by using the separation measure *S_DS_* proposed by Bara´basi et al. (30) (Equation 1). The smaller the value, the closer the two gene modules are in the background network (The principle of the calculation is shown in **Figure 2A**).

(1) $$S_{DS}=\langle d_{DS}\rangle -\frac{\langle d_{DD}\rangle +\langle d_{SS}\rangle }{2}$$

### 2.6 Network-based proximity analysis between HCC disease/TCM syndromes and drugs/formulae

**Figure 3:**
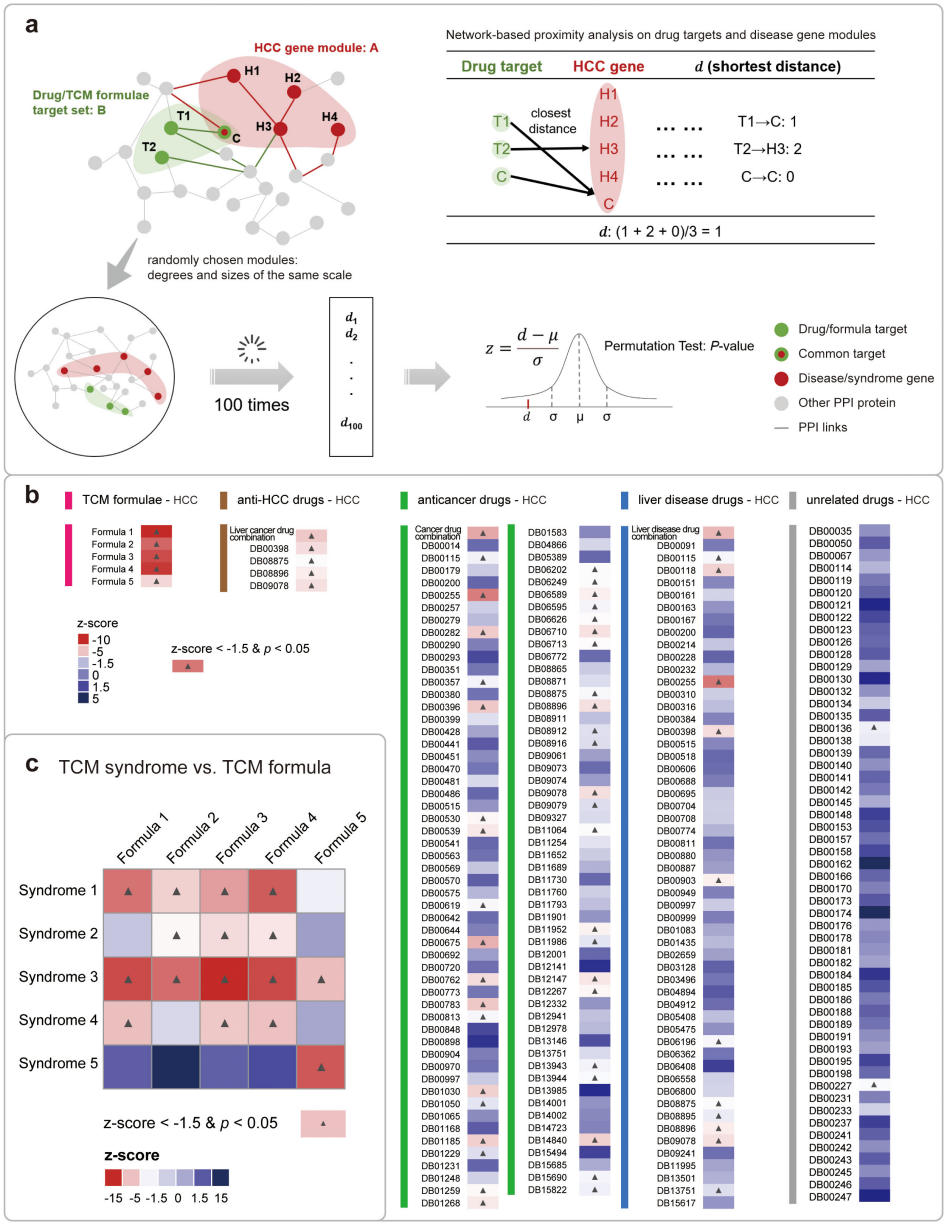
Network-based proximity analysis between HCC disease/TCM syndromes and drugs/formulae. **(A)** The detailed computation of the network-based proximity measure between genes of disease/TCM syndromes and that of drugs/formulae. **(B)** The analyzing results of the proximity measures between genes for HCC and that for 5 types of drugs/formulae. The colors of each cell indicate the relative proximity value in the *z-score*. And the colors of the bars stand in the left vertically stand for the drug type. And the cells with significant analyzing results were labeled with a black star. **(C)** The analyzing results of the proximity measures between 5 HCC syndrome and its 5 corresponding formulae. The figure is illustrated in a heatmap, with the meanings of the colors and stars similar to that in **(B)**.


We employed the disease-drug proximity measure proposed by Bara´basi et al. to quantify the network intersection between disease or TCM syndrome related protein set, and drug or formula related protein set (32). It is a method to calculate distances between two groups of nodes in the network while correcting for degree biases. In this work, the metric calculated the average shortest distance between the drug’s targets and the nearest disease protein on the PPI network, where H represents proteins for disease or TCM syndromes, and T represents drug/formula targets (Equation 2) (The principle of the calculation is shown in **Figure 3A**).

(2) $$d_{c}\left(H,T\right)=\frac{1}{\left\|T\right\|}\sum_{t\epsilon H}\min_{h\epsilon H}d\left(h,t\right)$$

Then, 100 random pairs of protein targets with the same size and same degree distribution were sampled from the whole network for the permutation test. Based on this, the *z-scores* and *P-values* for the proximity measures between drug-HCC disease, formula-HCC disease, and formula-TCM syndrome were calculated respectively (Equation 3). The criteria for significance is set to be *z-score<−1.5* and *P-value< 0.05* (33).


(3) $$z_{d_{HT}}=\frac{d_{HT}-\;\bar{d}}{\sigma }$$

where $\bar{d}$ and σ are the mean and standard deviation of the proximity measures based on 100 random samples.

The relevant computational codes for network separation and proximity measures can be accessed via the following GitHub repository: [Barabasi-Lab/COVID-19] (https://github.com/Barabasi-Lab/COVID-19).

### 2.7 KEGG-based functional analysis of genes related to diseases or drugs

For gene sets related to either HCC diseases, TCM syndromes, and formulae, we analyzed their potential functions through the Kyoto Encyclopedia of Genes and Genomes (KEGG) enrichment analysis. This analysis assesses the biological pathways of a particular gene set and was performed using the *clusterProfiler* R package with the following parameters: *pvalueCutoff* = 0.05, *pAdjustMethod* = “BH”, *qvalueCutoff* = 0.2, and the background gene set was the human gene set (34).

The R package used for KEGG enrichment analysis is clusterProfiler, version 4.10.1, available on GitHub at: [YuLab-SMU/clusterProfiler](https://github.com/YuLab-SMU/clusterProfiler).

### 2.8 Prioritization of active herbal ingredients for formulae based on random walking

We also prioritized the active herbal ingredients that constitute TCM herbs. Functionally related nodes tend to be close to each other in the network, which can be calculated based on the guilt-by-association method. In this study, we constructed a comprehensive heterogeneous network of drug-target-PPI, using a random walk with restart (RWR) algorithm (35), and took disease genes as seed nodes, to explore key ingredients in TCM formulae. We used ingredient targets or disease genes as mediators to connecting with the proteins constructing the PPI network. The TCM syndrome related genes were used as seed nodes for the random walk algorithms, and the probability of restart was set to 0.7. Networks were visualized using Gephi 0.9.3 (https://gephi.org/).

The RandomWalkRestartMH R package, version 1.22.0, was utilized for key molecule mining based on RWR methodology and can be found at: [alberto-valdeolivas/RandomWalkRestartMH] (https://github.com/alberto-valdeolivas/RandomWalkRestartMH).

### 2.9 Functional analysis of the prioritized herbal ingredients

In our previous work (20), we comprehensively curated the differentially expressed genes (DEGs) related to 211 ingredients after the cell line or animal models were perturbed by these ingredients. In this work, we firstly identified key ingredients for each formula based on network analysis, and then we connectively mapped these key ingredients to drugs based on the DEGs from both HERB and CMap databases (36), with the latter provided pharmacotranscriptomics dataset for modern drugs. As a result, the ingredients with unknown functions can be functional inferred based on the drugs that were connectively mapped to them with scores > 90. A score of 90 indicates that only 10% of reference perturbations showed stronger connectivity to the query, thus is recommended as the default threshold by CMap. In addition, we also conducted data mining on the key small molecules from the website of ClinicalTrials.gov to prove the druggability of these natural compounds.

## 3 Results

### 3.1 Five TCM syndromes of HCC and five recommended TCM formulae

According to the 2019 edition of the “*Guidelines for the Diagnosis and Treatment of Hepatocellular Carcinoma*”, issued by the National Health Commission of the Chinese government (10, 11), five distinct types of TCM syndromes were published and treated using five different TCM formulae. These syndromes have been categorized as common TCM classifications for HCC. The composition of these five formulae is shown in **Table 1**, with each formula corresponding to one of the five syndromes. The formulae involve a total of 44 herbs, and most of them appeared only once. And the information about their ingredients and targets is provided in the supplementary information (**Supplementary Appendix S1**, **Supplementary Table S1**, **Supplementary Figure S2**).

**Table 1:** Five recommended formulae for five TCM syndrome types of HCC.

TCM syndrome	Recommended formulae	Herb Latin name
Syndrome 1: liver depression and spleen deficiency	Formula 1: Modified Xiaoyao Powder and Sijunzi Decoction	*Codonopsis Radix, Atractylodis Macrocephalae Rhizoma, Poria, Persicae Semen, Bupleuri Radix, Angelicae Sinensis Radix, Paeoniae Radix Alba, Akebiae Caulis, Magnoliae Officinalis Cortex, Gardeniae Fructus, Curcumae Rhizoma, Glycyrrhizae Radix et Rhizoma*
Syndrome 2: liver-gallbladder dampness heat	Formula 2: Modified Yinchenhao Decoction	*Artemisiae Scopariae Herba, Gardeniae Fructus, Rhei Radix et Rhizoma, Lysimachiae Herba, Polyporus, Bupleuri Radix, Paeoniae Radix Alba, Curcumae Radix, Toosendan Fructus, Aurantii Fructus, Scutellariae Barbatae Herba, Paridis Rhizoma, Plantaginis Herba, Alismatis Rhizoma*
Syndrome 3: liver heat and blood stasis	Formula 3: Modified Longdan Xiegan Decoction and Xiayuxue Decoction	*Gentianae Radix et Rhizoma, Scutellariae Barbatae Herba, Gardeniae Fructus, Alismatis Rhizoma, Akebiae Caulis, Plantaginis Semen, Rehmanniae Radix, Bupleuri Radix, Persicae Semen, Curcumae Rhizoma, Rhei Radix et Rhizoma, Rubiae Radix et Rhizoma, Moutan Cortex, Glycyrrhizae Radix et Rhizoma*
Syndrome 4: spleen deficiency and dampness stagnation	Formula 4: Modified Sijunzi Decoction and Wupi Yin	*Astragali Radix, Codonopsis Radix, Atractylodis Macrocephalae Rhizoma, Poriae Cutis, Cyperi Rhizoma, Aurantii Fructus, Citri Reticulatae Pericarpium, Arecae Pericarpium, Benincasae Exocarpium, Alismatis Rhizoma, Coicis Semen, Solani Nigri Herba, Persicae Semen, Curcumae Rhizoma, Scutellariae Barbatae Herba, Glycyrrhizae Radix et Rhizoma*
Syndrome 5: liver-kidney yin deficiency	Formula 5: Modified Yiguanjian	*Rehmanniae Radix, Glehniae Radix, Ophiopogonis Radix, Angelicae Sinensis Radix, Lycii Fructus, Mori Fructus, Toosendan Fructus, Paeoniae Radix Rubra, Trionycis Carapax, Ligustri Lucidi Fructus, Ecliptae Herba, Moutan Cortex*

### 3.2 Genes for 5 TCM syndromes are topologically and functionally close to that for HCC

TCM syndromes are subtypes of a particular disease with a cluster of symptoms that can be stably observed in long-term clinical practice. In the HCC disease, 5 TCM syndromes were curated by national experts. We thus compared the molecular similarities among the HCC disease and its 5 different TCM syndromes respectively, based on their associated genes in the large human PPI network (**Figure 2A**). We firstly retrieved 2,835 syndrome-associated genes for five TCM syndrome types (**Supplementary Appendix S1**, **Supplementary Table S2**). Then we set out to further quantify the distances of the gene sets associated with HCC disease and five TCM syndromes by network-based separation analysis. The separation measure is formed as *S_DS_*, with smaller *S_DS_* indicating a tighter network-based overlap between two gene sets. It is shown that the mean separation scores were fairly small (0.10, green box in **Figure 2B**) when comparing the genes related to the HCC disease to genes for 5 TCM syndromes, indicating that the TCM syndromes of HCC are similar to the HCC disease in both phenotypes and molecular mechanisms (30). Furthermore, we also benchmarked this analysis by introducing random controls. To this end, we selected other 195 unrelated TCM syndrome types from SymMap database (**Supplementary Data S2**). We also computed the separation measures between genes of HCC and genes for these 195 other syndromes. And their mean separation scores are significantly larger (0.24, orange box in **Figure 2B**) than that for the comparison of HCC syndromes-related genes, indicating that diseases or TCM syndromes with similar clusters of symptoms will be closely related to adjacent network modules. To further demonstrate the robustness of the collected syndrome-related genes, we conducted the same calculations using different *P-value* thresholds. The results obtained were similar, to some extent confirming the reliability of the syndrome-related genes identified through SymMap mining in the context of traditional Chinese medicine (**Supplementary Appendix S1**, **Supplementary Figure S3**).

In addition to the topological approach, we further compared HCC and its related TCM syndromes based on the functional analysis of genes. We performed functional enrichment analysis on the HCC disease/HCC syndromes gene sets based on the KEGG annotation, and found 35 classes of enriched metabolic pathways for these genes (**Figure 2C**). And we showed that the associated pathway for the HCC disease overlapped with that for the five TCM syndrome types of HCC. Among them, syndrome 1 (syndrome of liver depression and spleen deficiency) demonstrated the highest degree of overlap with HCC. By integrating clinical data, some researchers have found that this syndrome is one of the most common syndromes of liver disease (37, 38). Meanwhile, the integrated application of intestinal microbiota and metabolomics research further provides experimental basis for the efficacy of TCM formulae in the treatment of hepatocellular carcinoma of the syndrome type (39). In addition, each syndrome overlapped to the HCC disease in a different set of pathways, indicating that the differentiation of a disease into several TCM syndrome subtypes captures the different aspects of the disease at the molecular level. The perspective promoted us to further untangle the molecular basis of different TCM formulae that were used to treat different HCC syndromes.

### 3.3 Genes for 5 TCM formulae are topologically and functionally close to that for HCC and its TCM syndromes

Different from modern drugs, TCM formula is consisting of multiple herbs, with each herb constituted by multiple ingredients. The mixture nature of TCM formula brought huge difficulties in studying its mechanism of action. Fortunately, with the introduction of systems biology as well as our previous study of the HERB database (20), TCM formulae can be connected to gene targets in recent years based on the global viewpoints of network medicine (40). We thus gathered potential gene targets for the 5 TCM formulae of HCC, and then compared them with the genes for HCC and its TCM syndromes in the large human PPI network. We used the network-based proximity measure to compute the closest distance between a gene set related to drugs/formulae and that for disease/TCM syndromes (**Figure 3A**), as the metric is shown to be suitable for quantifying treatment effect (32). Inspired by several studies using this metric in drug discovery and drug repurposing, we expected that effective drugs would demonstrate more close gene modules to the disease than irrelevant drugs (33, 41–43).

Similarly, we benchmarked the analyzing results for TCM formulae by introducing other drugs that are approved for treating several disease types, including HCC, liver disease, and cancers. We performed the network-based proximity analysis for several pairwise comparisons of gene sets. For example, TCM formulae *vs.* HCC, anti-HCC drugs *vs.* HCC, other anti-cancer drugs *vs.* HCC, other liver disease drugs *vs.* HCC, and drugs unrelated to either liver disease or cancer *vs.* HCC. For each comparison, we conducted permutation test to calculate the relative *z-score* values and *P-values*, and defined the statistically significant pairs when it meets a stringent criteria: *z-score* <−1.5 and *P-value* < 0.05 (33). As a result, we found that four first- and second-line anti-HCC drugs, named sorafenib (DB00398, *z-score* = -4.27), cabozantinib (DB08875, *z-score* = -3.28), regorafenib (DB08896, *z-score* = -3.79), and lenvatinib (DB09078, *z-score* = -4.18), all showed good proximity to HCC, which is consistent with their clinical indications (2). In contrast, other types of drugs demonstrated a fairly decreased ratio of significant proximal gene modules to HCC. As expected, the proximity analyzing results for five TCM formulae are all statistically significant (**Figure 3B**). Based on these findings, we expanded our scope of analysis to include direct comparisons with other database resources. This benchmarking process allowed us to further validate the effectiveness of our methodology. Different formula-target data from two reputable high-quality databases, including HIT 2.0 (44) and ITCM (45), provided a solid foundation for our network-based proximity analysis. We then proceeded to conduct a series of pairwise comparisons of the genomes, employing the same stringent statistical significance criteria previously mentioned. Our comparative analysis revealed that the results from different data sources were largely consistent overall. In terms of topological analysis (**Supplementary Appendix S1**, **Supplementary Figure S4**, **Supplementary Data S5**), the targets of TCM formulas, as extracted from these databases, exhibited significant proximity to HCC genes, underscoring the inherent capacity of TCM to engage with the intricate pathophysiology of HCC. This topological congruence across different data sources not only validates the consistency of our results but also reaffirms the systemic approach inherent to TCM. The holistic nature of TCM is reflected in its ability to address HCC through the modulation of multiple biological pathways concurrently. This polypharmacological approach is particularly pertinent in the context of complex diseases like HCC, where a multi-target intervention strategy may offer superior therapeutic outcomes compared to single-target treatments. This result demonstrated the efficiency of TCM formulae in treating HCC, which aligns with the national guidelines for the treatment of HCC in China.

Next, we examined the proximity metrics between each TCM syndrome and its corresponding TCM formula (**Figure 3C**). It is showed that all five recommended syndrome-formula pairs (cells on the main diagonal in **Figure 3C**) exhibiting significant proximity, further proved their clinical efficacy. Besides, among them, the syndrome 5 is exclusively associated with formula 5, implying that the dysregulated state of syndrome 5 is distinct from other syndromes in the biological network. A clinical study utilized peripheral blood mononuclear cells from HCC patients as test specimens and identified differences between the liver-kidney yin deficiency syndrome group and the non-liver-kidney yin deficiency syndrome group through bioinformatics analysis. The result further supports our findings (46). That is to say, the molecular analysis of the TCM clinical experiences across millennia can shed light on better precision medicine in the future.

**Figure 4:**
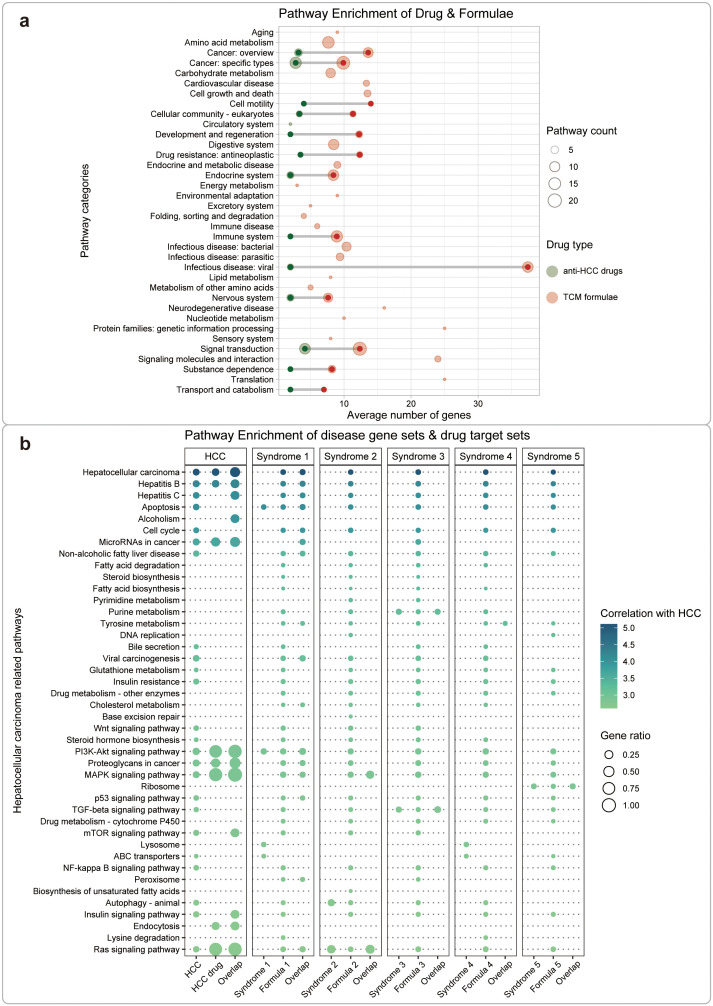
Functional analysis between HCC disease/TCM syndromes and drugs/formulae. **(A)** Comparison of regulated pathways between anti-HCC drugs and TCM formulae based on the KEGG annotation. These pathways were clustered into 36 classes according to KEGG orthology. The color of the bubbles represents different types of drugs, red for TCM formulae and green for small molecule drugs. The size of the bubbles represents the number of the same type of pathway. **(B)** Comparison of HCC-related pathways regulated by anti-HCC drugs and TCM formulae. We performed KEGG enrichment analysis for gene sets related to HCC, its TCM syndromes, drugs, or TCM formulae, respectively. We also performed KEGG enrichment analysis for gene sets concurrently related to the disease-drug or the syndrome-formula pairs. The size of the bubbles represents the ratio between the relevant genes and the set of pathway genes. The color of the bubbles represents the correlation between the pathway and HCC. Based on PubMed searching, the literature records were retrieved using the keywords “hepatocellular carcinoma” & “pathway name”. The log-transformed number of such papers was used as a measure for the correlation between HCC and the pathway. Finally, we screened and displayed the top 10% pathways (42 pathways) most relevant to HCC.

We then performed KEGG enrichment analysis on gene sets related to HCC, its 5 TCM syndromes, drugs, and formulae (**Supplementary Appendix S1**, **Supplementary Figure S5**). We found that the gene targets for the TCM formulae for HCC shared several common KEGG pathways with that for anti-HCC drugs, including cancer: specific types, signal transduction, cancer: overview, endocrine system (**Figure 4A**). Compared with modern drugs, TCM formulae targeted larger number of pathways, not only includes cancer-related pathway but also embraces other pathways, such as viral infectious disease. This result is interpretable because HCC frequently originated from viral infections, including hepatitis B virus (HBV) and hepatitis C virus (HCV) (47). In contrast, small molecule drugs are more focused on cancer-specific pathways and signal transduction processes. These results are consistent with the target-based drug discovery mode of small molecule drugs (48) and phenotypic drug discovery for TCM formula (17). This consensus is further reinforced by the convergence of findings from diverse sources, which not only validates the robustness of our results but also underscores the potential of integrating systems biology with TCM (**Supplementary Appendix S1**, **Supplementary Figure S5**).

Furthermore, we analyzed the related pathways for HCC, and its TCM syndromes, formulae, and drugs in detail, based on 42 curated pathways mined from the PubMed database (**Figure 4B**). We then performed KEGG enrichment analysis for gene sets related to HCC, its TCM syndromes, drugs or TCM formulae, respectively. We also performed KEGG enrichment analysis for gene sets concurrently related to the disease-drug or the syndrome-formula pairs. The results demonstrated similar patterns as that in **Figure 4A**. Small molecule drugs showed fewer but more specific related pathways, while TCM formulae seem to act on more broad set of regulated pathways. We also noticed that different TCM formulae showed distinct major regulated pathways. For example, formula 1 regulates similar pathways with small molecule drugs, formula 2 mainly targets MAPK and RAS signaling pathways, formula 3 specifically targets purine metabolism and TGF-beta signaling pathways, formula 4 exclusively targets tyrosine metabolism, and formula 5 only targets ribosome-related processes. The results further provide functional evidence for understanding the usage of different treatment material for each detailed differentiated subtype of disease in a traditional TCM way.

### 3.4 The prioritization of active herbal ingredients in different formulae

Furthermore, we intended to dissect active small molecules in TCM formulae, in order to elucidate the key material basis from the mixture and provide support for modern drug discovery. To this end, we assembled a new heterogeneous network covering herbs, ingredients, and protein interactions by integrating the PPI network to the HERB target curations (**Figure 1D**). Then we navigated the network to explore its global topology by using the random walk with restart (RWR) algorithm (35). In a systematic effort to validate and demonstrate the advantages of our approach over traditional network pharmacology computational methods, we compared our drug screening algorithm with these methods. This systematic comparison was conducted to evaluate the efficacy and accuracy of the RWR algorithm in identifying the most relevant ingredients within the TCM formulae. The detailed results of the systematic comparison can be found in the supporting information (**Supplementary Appendix S1**, **Supplementary Figure S6**).

**Figure 5:**
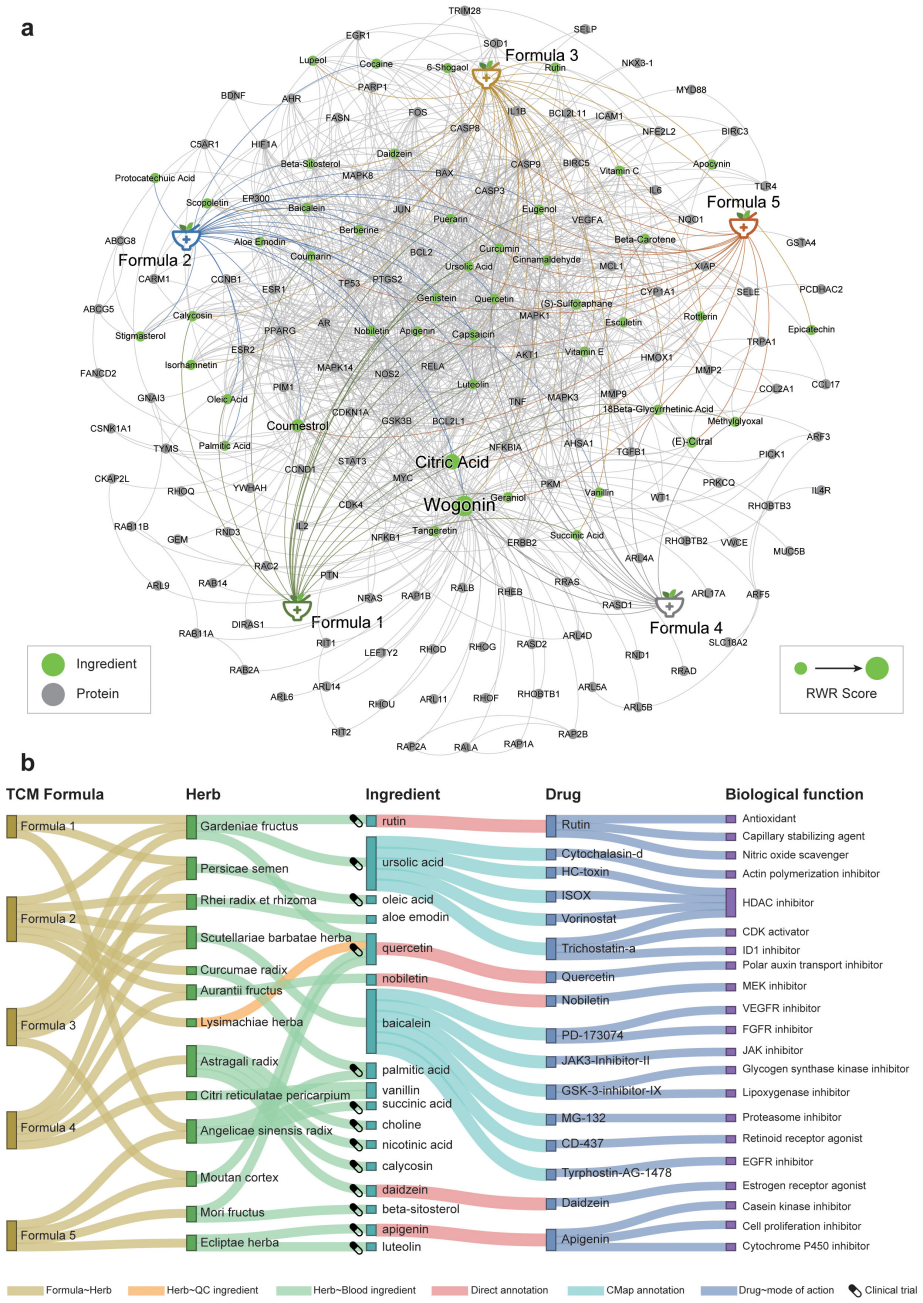
The prioritization of herbal ingredients to intervene in different HCC-related TCM syndromes. **(A)** The heterogeneous network consists of formulae, ingredients, and genes. We only showed the top 1% RWR score nodes in the network. The font size is positively correlated with the mean random walk score of the five formulae. **(B)** Functional annotation of active herbal ingredients. The ingredients for each herb either can be absorbed into the blood circulation or can be used for quality inspection. The clinical trial icons show on the left of each ingredient indicate that there were clinical trial records for this ingredient on ClinicalTrials.gov, strong evidence for this ingredient as potential drug.

We used genes of the HCC-related TCM syndromes as the seed nodes to perform the RWR algorithm, and then measured the association degree of each ingredient in the heterogeneous network. Finally, the functions of top predicted ingredients were explored based on literature as well as connectivity mapping analysis. The 46 key ingredients were provided in **Table 2**. The networks of these ingredients with the TCM formulae and syndromes were shown in **Figure 5A**.

**Table 2:** The key ingredients of the formulae for intervening five TCM syndromes.

Ingredient ID	Ingredient name	Source	Ingredient ID	Ingredient name	Source
HBIN020984	citric acid	Formula 1,2,3,4,5	HBIN048047	vitamin c	Formula 3,5
HBIN041495	quercetin	Formula 1,2,3,4,5	HBIN019762	beta-carotene	Formula 4,5
HBIN021608	coumarin	Formula 1,2,3,4,5	HBIN040908	protocatechuic acid	Formula 2
HBIN021985	curcumin	Formula 1,2,3,4,5	HBIN021188	cocaine	Formula 2
HBIN019690	capsaicin	Formula 1,2,3,4	HBIN017893	berberine	Formula 2
HBIN026067	eugenol	Formula 1,2,3,5	HBIN017508	baicalein	Formula 2
HBIN047613	ursolic acid	Formula 1,2,3,5	HBIN038680	palmitic acid	Formula 2
HBIN027456	genistein	Formula 1,2,3	HBIN047744	vanillin	Formula 2
HBIN002095	18beta-glycyrrhetinic acid	Formula 1,3,4	HBIN044918	stigmasterol	Formula 2
HBIN048051	vitamin e	Formula 1,3,4	HBIN037171	nobiletin	Formula 2
HBIN045062	succinic acid	Formula 1,3,4	HBIN042670	rutin	Formula 3
HBIN027528	geraniol	Formula 1,3,5	HBIN033751	lupeol	Formula 3
HBIN016408	apigenin	Formula 1,2,5	HBIN016505	apocynin	Formula 3
HBIN035246	methylglyoxal	Formula 1,4,5	HBIN042467	rottlerin	Formula 3
HBIN048372	wogonin	Formula 2,3,4	HBIN025287	epicatechin	Formula 3
HBIN020653	cinnamaldehyde	Formula 2,3,4	HBIN031114	isorhamnetin	Formula 3
HBIN010721	(S)-sulforaphane	Formula 1,4	HBIN025796	esculetin	Formula 3
HBIN033803	luteolin	Formula 1,2	HBIN043445	scopoletin	Formula 3
HBIN038026	oleic acid	Formula 1,2	HBIN015286	aloe emodin	Formula 3
HBIN019425	calycosin	Formula 1,3	HBIN045469	tangeretin	Formula 4
HBIN041253	puerarin	Formula 1,3	HBIN024822	(e)-citral	Formula 5
HBIN018278	beta-sitosterol	Formula 2,3	HBIN012818	6-shogaol	Formula 5
HBIN021620	coumestrol	Formula 2,5	HBIN022577	daidzein	Formula 5

Several ingredients were prioritized repeatedly in five formulae, such as citric acid, quercetin, coumarin, and curcumin. Among them, citrate is an important metabolic regulator, which is involved in inflammation and cancer (49, 50). It is shown that citrate uptake broadly affects tumor cell metabolism through citrate-dependent metabolic pathways (51), because citrate promotes lipid excess biosynthesis and disrupts lipid metabolism in cancer cells, resulting in cancer cell senescence and growth inhibition (52). Curcumin and quercetin not only have inhibitory effects on HCC (53, 54), but also have the potential to interfere with HCC comorbidities. Curcumin can also inhibit HCV entry of all major genotypes into primary human hepatocytes, thereby impairing viral binding and fusion (55). Quercetin also exhibited hepatoprotective effects on acute hepatitis by inhibiting the TRAF6/JNK pathway (56). To substantiate the reliability of our results about the small molecules screened, we also have conducted an extensive review of the most recent experimental literature, encompassing a variety of *in vitro* cellular assays and *in vivo* animal studies (**Supplementary Appendix S1**, **Supplementary Table S3**). This comprehensive analysis has provided a wealth of evidence supporting the efficacy of the compounds we have identified. These results demonstrated the power of our network medicine analysis for decoding promising material basis for TCM.

Some ingredients have evidence that they were absorbed into the blood or used for quality inspection based on SymMap annotation (17). Therefore, these ingredients are more likely to be the pharmacological substances of the formulae. So, we functionally annotated these ingredients based on their pharmacotranscriptomics datasets curated in the HERB database (**Figure 5B**). The results showed more evidence for their potential pharmacological effects. For example, nobiletin from *Aurantii Fructus* is a MEK inhibitor, which has been shown to significantly inhibit HCC *in vitro* and *in vivo* (57), by regulating cell proliferation, differentiation, angiogenesis, and survival (58). Note that the first-line drug, sorafenib, exerted its anti-HCC effects by targeting the RAF/MEK/ERK pathway (59). Besides, baicalein, a flavonoid from *Scutellariae barbatae herba*, may act as VEGFR inhibitor, FGFR inhibitor, and JAK inhibitor, which were significantly associated with anti-HCC effects (60). Note that VEGFR is the primary target of most FDA-approved HCC drugs such as sorafenib, lenvatinib, and regorafenib. We also utilized RDKit to visualize the structural similarities between nobiletin, baicalein, and sorafenib (**Supplementary Appendix S1**, **Supplementary Figure S7**). The result revealed that baicalein exhibits a higher degree of structural similarity to the core scaffold of sorafenib. This finding suggests that baicalein and sorafenib may share molecular fragments that are crucial for their pharmacological actions, potentially indicating a common mode of interaction with biological targets. Although nobiletin showed a lower overall similarity to sorafenib, the visualization provided by the similarity maps was invaluable. It shed light on the specific molecular features that contribute to the observed similarity, offering a roadmap for the discovery of new structural scaffolds with potential therapeutic relevance. Therefore, baicalein may exert its anti-angiogenic effect as a new VEGFR inhibitor in HCC. In conclusion, these active herbal ingredients exert diverse pharmacological activities in treating different TCM syndromes of HCC, and ultimately jointly intervene in the progression of HCC.

## 4 Discussion

The term “syndrome” is a pathological summary of a particular stage in the development of a disease within the body. It encapsulates the location, etiology, nature, and relationship between pathogenic and healthy factors, accurately reflecting the essence of pathological changes at a specific stage of the disease development process. In comparison to symptoms, it offers a more comprehensive and integrated understanding of the nature of the disease. Functionally, it aids in the physiological and pathological understanding of phenomena, guiding traditional Chinese medicine in the recognition of disease manifestations and occurrences. It serves as the primary basis for understanding diseases and syndrome differentiation in traditional Chinese medicine. Modern medicine shares many viewpoints with traditional medicine. In terms of understanding health and disease, modern medicine similarly emphasizes a fundamental focus on the material aspect, employing a dialectical and unified approach to prevention and treatment. It recognizes the interconnected principles that govern qualitative changes in disease development. By exploring the expression of syndrome genes—especially in cases of the same disease with different patterns or different diseases with similar patterns—our aim is to reveal all genes and their functions related to a specific pattern. This aims to elucidate the essence of the syndrome from the overall level of gene expression. Conversely, modern medical research indicates that the same disease can exhibit completely different patterns of gene expression, while different diseases may manifest similar patterns of gene expression. This aligns with the theory in traditional Chinese medicine, where the same disease may present different patterns, and different diseases may share similar patterns.

As a complementary therapy of modern medicine, TCM has a unique effect on the intervention of complex diseases. In the clinical practice of TCM, the identification of syndromes by the four ways of TCM diagnosis including looking, listening, asking, and feeling the pulse is the core to TCM in clinical practice, since the syndromes are the main basis for prescribing in TCM. As syndromes are disease terms unique to Chinese medicine, it has been impossible for a long time to apply biological terms to characterize these syndromes. The past TCM network medicine studies were mostly based on the biological network of modern diseases, which may not fully conform to the theoretical basis of TCM. In previous work, we constructed associations between TCM syndromes and disease genes by mapping modern phenotypes to TCM symptoms. Therefore, in this work, based on TCM syndrome-related gene sets of the HCC disease, we explored the biological basis of the intervention of five TCM syndrome types by different formulae and screened potential key herbs and their ingredients through topological analysis and functional mining.

Diseases were viewed as perturbed states of molecular network systems (61). Environmental factors, genetic factors, and drugs are thought to interfere with this system (62, 63). Unlike modern medicine, TCM usually intervenes in this system by balancing the whole state of the human body. It is an important task to understand the underlying mechanism of the detailed classification of diseases into TCM syndromes and treat each of them by different formulae. In this work, we used network-based separation metric and functional enrichment analysis to reveal that certain degrees of similarity exists between the HCC disease and 5 TCM syndrome types. The results of this work partly clarify the potential rationality of TCM syndrome and provide its scientific basis. Next, we found that the gene modules related to these syndromes and their recommended formulae were significantly adjacent. Moreover, compared with unrelated formulae, the proximity measures of recommended formulae are generally higher, further supporting the necessity of the syndrome-formula relationships and the effectiveness of formula treatment syndromes in TCM. From a functional view, the action mode of western medicine is more specific, while the action mode of TCM is more comprehensive. Specifically, small molecule drugs treat HCC by inhibiting/blocking certain signal transduction events, while TCM formulae could intervene in HCC from neuro-immune-endocrine pathways. The results were consistent with the target-based discovery of small-molecule drugs, and the phenotypic-based discovery of TCM herbs and their combinations as formulae.

Using the biological network analysis methods employed in this study, we examined the disease mechanisms of HCC from both the genetic and protein perspectives, applying the viewpoints of both TCM and Western medicine. Our analysis revealed that, although TCM and Western medicine have different diagnostic and treatment models, they share similar sets of disease-related genes at the genetic and protein levels, forming comparable disease pathways. Simultaneously, we identified that different treatment strategies, whether in the form of TCM formulae or small molecules, can effectively intervene in HCC, albeit with specificity in their target pathways, contingent on various TCM subtypes. The proximity calculations between “formula-syndrome” pairs in our research reveal the potential for personalized TCM treatments. By understanding the molecular underpinnings of TCM syndromes, we can customize TCM treatment plans to individual patients, selecting or modifying formulas to match their unique conditions. This approach exemplifies the potential of precision medicine within the framework of TCM and offers a more precise and effective treatment strategy.

Finally, our research has the capacity to predict core natural compounds required for HCC treatment, and find the core ingredients of five formulae, such as citric acid, quercetin, coumarin, and curcumin. The history of drug discovery from traditional medicines, including success stories like ephedrine, artemisinin, and tetrahydropalmatine, inspires us. The active ingredients identified in our study, such as nobiletin and baicalein, offer promising leads for drug development. These compounds have been extensively studied for their *in vitro* and *in vivo* anti-HCC effects, reinforcing the value of bioactive compounds discovery from TCM. In the future, our intention is to build upon the current findings with targeted experiments that will further elucidate the mechanisms of action and potential clinical applications of the TCM formulas and compounds in question. We are particularly interested in exploring the synergistic effects of these small molecules when used in combination to combat HCC.

In conclusion, our study offers valuable insights into the treatment of HCC, aligning traditional Chinese medicine with modern clinical practices and providing a solid foundation for the integration of TCM into HCC management. These results also inspired us to further decode the valuable TCM knowledge for modern drug discovery.

## 5 Conclusions

In this work, we first demonstrated that the gene sets associated with five TCM syndrome types are topologically and functionally close to the HCC disease’s genes. Next, the intervention mechanisms of the five syndrome types were analyzed at three different levels: formulae, herbs, and ingredients. In contrast to small molecule drugs, TCM formulae intervene in syndromes through a multi-component, multi-target and multi-pathway network modulation. Priorities of ingredients that can be confirmed by the literature were also uncovered. The results of multilevel comparisons provide some evidence of the scientific validity of using different formulae for different syndrome types of HCC. In conclusion, this work provides a methodological reference for the systematic study of the same disease with different syndrome patterns intervention with TCM formulae, which is of great significance to the modern research of TCM therapy.

## Data Availability

The original contributions presented in the study are included in the article/**Supplementary Material**. Further inquiries can be directed to the corresponding authors.
