# Supplementary material for: Network medicine analysis for dissecting the therapeutic mechanism of consensus TCM formulae in treating hepatocellular carcinoma with different TCM syndromes
Source: Front Endocrinol (Lausanne). 2024 Aug 15;15:1373054. doi: 10.3389/fendo.2024.1373054 (PMC11357915; doi:10.3389/fendo.2024.1373054)
Supplement: Supplementary file 1 [file DataSheet1.docx]

**Supplementary Information for:**

**Network medicine analysis for dissecting the** **therapeutic mechanism** **of consensus TCM formulae in treating hepatocellular carcinoma with different TCM syndromes**

Kai Gao^1, †^, WanChen Cao^1, †^, ZiHao He^1^, Liu Liu^1^, JinCheng Guo^1^, Lei Dong^1^, Jini Song^2^, Yang Wu^3, *^, Yi Zhao^1, 3, *^

1 School of Traditional Chinese Medicine, Beijing University of Chinese Medicine, Chaoyang District, Beijing 100029, China

2 New York Institute of Technology College of Osteopathic Medicine, Arkansas State University, Jonesboro, Arkansas, United States

3 The Research Center for Ubiquitous Computing Systems (CUbiCS), Institute of Computing Technology, Chinese Academy of Sciences, Beijing 100190, China

* To whom correspondence should be addressed.

Tel: +86-15120098608, Email: [wuyang@ict.ac.cn](mailto:wuyang@ict.ac.cn) (Yang Wu);

Tel: +86 10 6260 0822, Fax: +86 10 6260 1356, Email: [biozy@ict.ac.cn](mailto:biozy@ict.ac.cn) (Yi Zhao).

† The authors wish it to be known that, in their opinion, the first two authors should be regarded as Joint First Authors.

**This PDF file includes:**

Supplementary text

Figures S1 to S7

Tables S1 to S3

Legends for Supplementary Data 1 to Data 5

**Other supplementary materials for this manuscript include the following:**

Supplementary Data 1 to Data 5

**1. Screening of active small molecules based on QED metric**

The QED stands for quantitative estimation of drug-likeness. The empirical rationale of the QED measure reflects the underlying distribution of molecular properties including molecular weight, logP, topological polar surface area, number of hydrogen bond donors and acceptors, the number of aromatic rings and rotatable bonds, and the presence of unwanted chemical functionalities. We calculated QED values for all approved small molecule drugs in the drugbank database using the RDKit python package. These values conformed to a normal distribution, so a screening threshold of 0.10 was determined based on the 5% quantile to ensure that 95% of small molecule drugs could be selected (**Figure. S1**).


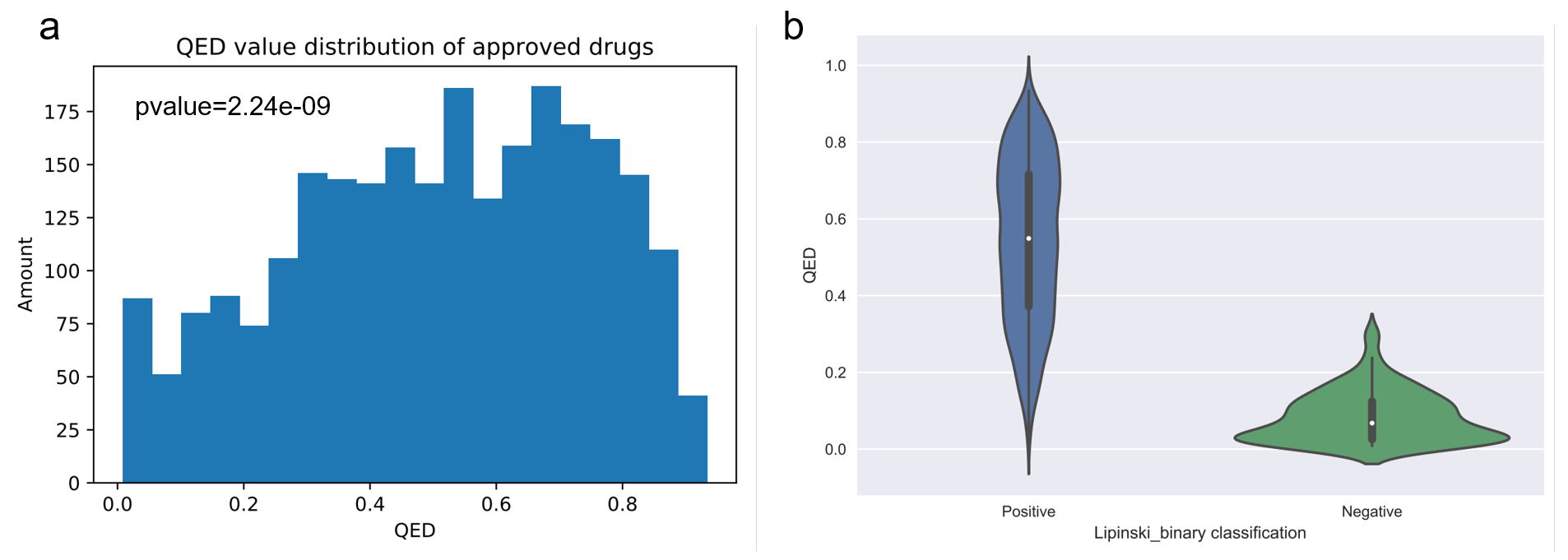


**Figure. S1 Determine the QED threshold.** **a**) Distribution of QED values for approved small molecule drugs. **b**) Dichotomous distribution of QED values according to Lipinski's rule. Lipinski rule: MolWt(m) <= 500, MolLogP(m) <= 5, CalcNumLipinskiHBA(m) <= 10, CalcNumLipinskiHBD(m) <= 5. When both conditions are met at the same time, it is positive, otherwise it is negative. The QED values of negative drugs were distributed around 0.1.

**2. Retrieving gene sets of five TCM formulae and their herbs**

We retrieved and assembled gene sets of TCM formulae and their herbs in the guidelines based on the HERB database (**Table S1**).

**Table S1 Recommended Formulae information for different TCM syndromes collected from Herb database**

| Syndrome | Recommended formula | Herb pinyin name | Herb Latin name | Ingredient count | Target count |
| --- | --- | --- | --- | --- | --- |
| Syndrome 1  syndrome of liver depression and spleen deficiency | Formula 1  Modified Xiaoyao Powder and Sijunzi Decoction | Dang Shen | Codonopsis Radix | 105 | 1100 |
|  |  | Bai Zhu | Atractylodis Macrocephalae Rhizoma | 64 | 933 |
|  |  | Fu Ling | Poria | 52 | 237 |
|  |  | Tao Ren | Persicae Semen | 49 | 308 |
|  |  | Chai Hu | Bupleuri Radix | 226 | 1261 |
|  |  | Dang Gui | Angelicae Sinensis Radix | 111 | 371 |
|  |  | Bai Shao | Paeoniae Radix Alba | 75 | 485 |
|  |  | Mu Tong | Akebiae Caulis | 48 | 164 |
|  |  | Hou Po | Magnoliae Officinalis Cortex | 97 | 854 |
|  |  | Zhi Zi | Gardeniae Fructus | 81 | 704 |
|  |  | E Zhu | Curcumae Rhizoma | 43 | 192 |
|  |  | Gan Cao | Glycyrrhizae Radix Et Rhizoma | 165 | 440 |
| Syndrome 2  syndrome of liver-gallbladder dampness heat | Formula 2  Modified Yinchenhao Decoction | Yin Chen | Artemisiae Scopariae Herba | 50 | 548 |
|  |  | Zhi Zi | Gardeniae Fructus | 81 | 704 |
|  |  | Da Huang | Rhei Radix Et Rhizoma | 53 | 1048 |
|  |  | Jin Qian Cao | Lysimachiae Herba | 59 | 259 |
|  |  | Zhu Ling | Polyporus | 19 | 35 |
|  |  | Chai Hu | Bupleuri Radix | 226 | 1261 |
|  |  | Bai Shao | Paeoniae Radix Alba | 75 | 485 |
|  |  | Yu Jin | Curcumae Radix | 142 | 1063 |
|  |  | Chuan Lian Zi | Toosendan Fructus | 54 | 2066 |
|  |  | Zhi Ke | Aurantii Fructus | 19 | 133 |
|  |  | Ban Zhi Lian | Scutellariae Barbatae Herba | 64 | 679 |
|  |  | Chong Lou | Paridis Rhizoma | 3 | 78 |
|  |  | Che Qian Cao | Plantaginis Herba | 88 | 424 |
|  |  | Ze Xie | Alismatis Rhizoma | 22 | 126 |
| Syndrome 3  syndrome of liver heat and blood stasis | Formula 3  Modified Longdan Xiegan Decoction and Xiayuxue Decoction | Long Dan | Gentianae Radix Et Rhizoma | 37 | 229 |
|  |  | Ban Zhi Lian | Scutellariae Barbatae Herba | 64 | 679 |
|  |  | Zhi Zi | Gardeniae Fructus | 81 | 704 |
|  |  | Ze Xie | Alismatis Rhizoma | 22 | 126 |
|  |  | Mu Tong | Akebiae Caulis | 48 | 164 |
|  |  | Che Qian Zi | Plantaginis Semen | 31 | 297 |
|  |  | Di Huang | Rehmanniae Radix | 8 | 29 |
|  |  | Chai Hu | Bupleuri Radix | 226 | 1261 |
|  |  | Tao Ren | Persicae Semen | 49 | 308 |
|  |  | E Zhu | Curcumae Rhizoma | 43 | 192 |
|  |  | Da Huang | Rhei Radix Et Rhizoma | 53 | 1048 |
|  |  | Qian Cao | Rubiae Radix Et Rhizoma | 30 | 132 |
|  |  | Mu Dan Pi | Moutan Cortex | 49 | 542 |
|  |  | Gan Cao | Glycyrrhizae Radix Et Rhizoma | 165 | 440 |
| Syndrome 4  syndrome of spleen deficiency and dampness stagnation | Formula 4  Modified Sijunzi Decoction and Wupi Yin | Huang Qi | Astragali Radix | 71 | 1032 |
|  |  | Dang Shen | Codonopsis Radix | 105 | 1100 |
|  |  | Bai Zhu | Atractylodis Macrocephalae Rhizoma | 64 | 933 |
|  |  | Fu Ling Pi | Poriae Cutis | 37 | 226 |
|  |  | Xiang Fu | Cyperi Rhizoma | 74 | 292 |
|  |  | Zhi Ke | Aurantii Fructus | 19 | 133 |
|  |  | Chen Pi | Citri Reticulatae Pericarpium | 64 | 571 |
|  |  | Da Fu Pi | Arecae Pericarpium | 35 | 432 |
|  |  | Dong Gua Pi | Benincasae Exocarpium | 76 | 296 |
|  |  | Ze Xie | Alismatis Rhizoma | 22 | 126 |
|  |  | Yi Yi Ren | Coicis Semen | 28 | 467 |
|  |  | Long Kui | Solani Nigri Herba | 42 | 897 |
|  |  | Tao Ren | Persicae Semen | 49 | 308 |
|  |  | E Zhu | Curcumae Rhizoma | 43 | 192 |
|  |  | Ban Zhi Lian | Scutellariae Barbatae Herba | 64 | 679 |
|  |  | Gan Cao | Glycyrrhizae Radix Et Rhizoma | 165 | 440 |
| Syndrome 5  syndrome of liver-kidney yin deficiency | Formula 5  Modified Yiguanjian | Di Huang | Rehmanniae Radix | 8 | 29 |
|  |  | Bei Sha Shen | Glehniae Radix | 61 | 540 |
|  |  | Mai Dong | Ophiopogonis Radix | 5 | 72 |
|  |  | Dang Gui | Angelicae Sinensis Radix | 111 | 371 |
|  |  | Gou Qi Zi | Lycii Fructus | 135 | 1698 |
|  |  | Sang Shen | Mori Fructus | 74 | 1654 |
|  |  | Chuan Lian Zi | Toosendan Fructus | 54 | 2066 |
|  |  | Chi Shao | Paeoniae Radix Rubra | 73 | 690 |
|  |  | Bie Jia | Trionycis Carapax | 4 | 34 |
|  |  | Nv Zhen Zi | Ligustri Lucidi Fructus | 115 | 2516 |
|  |  | Mo Han Lian | Ecliptae Herba | 25 | 524 |
|  |  | Mu Dan Pi | Moutan Cortex | 49 | 542 |

**3. Herbs composition of the recommended formulae**

We separately conducted a statistical analysis of the herb overlaps between these formulae (**Figure. S2**), and it's not difficult to observe varying degrees of overlap among the constituting of these formulae. These overlaps may indicate the presence of some similarities in the treatment mechanisms of the formulae.


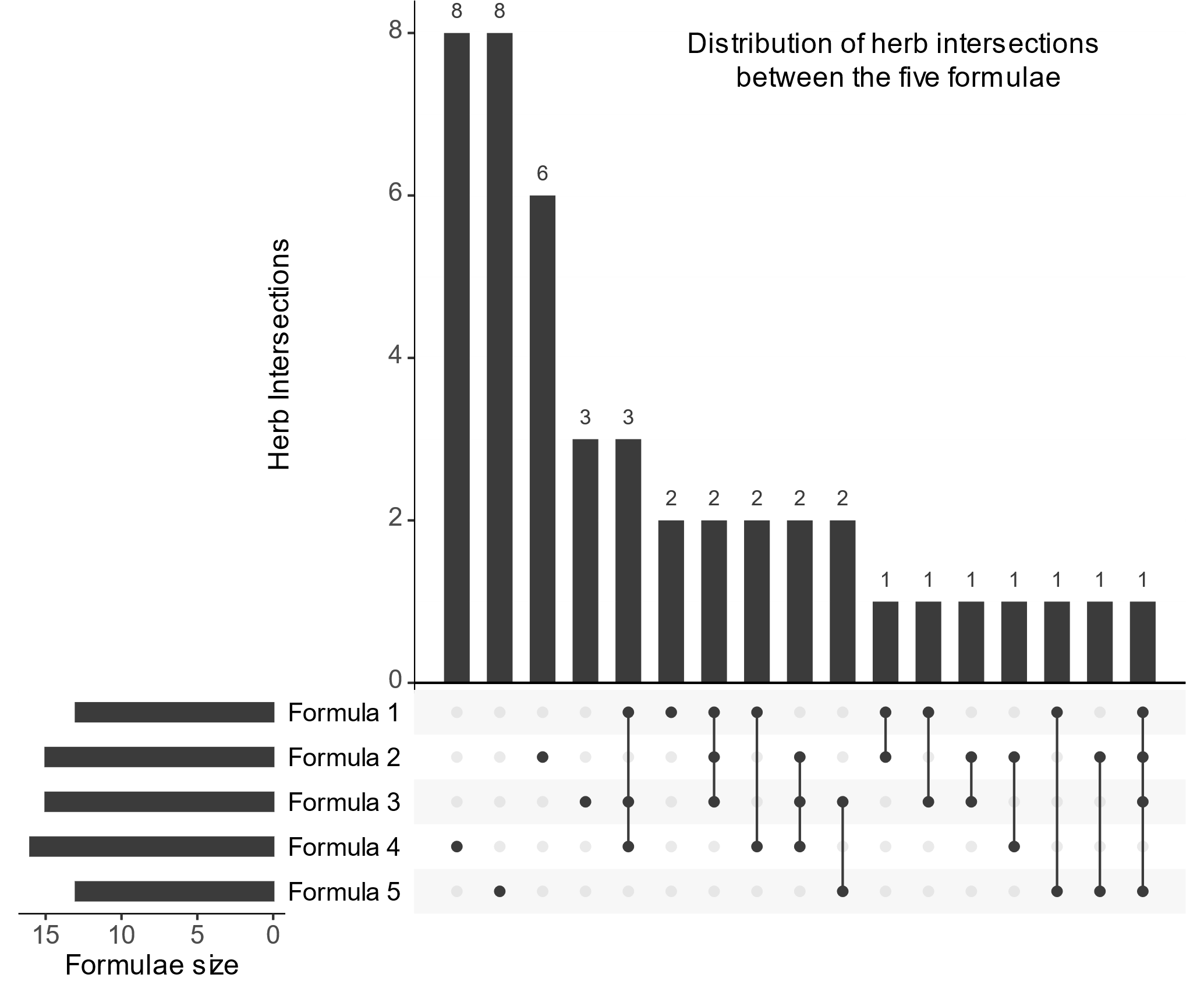


**Figure. S2 Herb overlap in recommended formulae.** Statistical analysis of herb overlaps among the five recommended formulae.

**4. Retrieving gene sets of five TCM syndromes and their symptoms**

We retrieved and assembled gene sets of five TCM syndromes and their symptoms of hepatocellular carcinoma (HCC) in the guidelines based on the SymMap database. Among them, 22 related TCM symptom gene sets were queried (**Table S2**).

**Table S2 TCM symptoms corresponding to different TCM syndromes collected from the SymMap database**

| Syndrome ID | Syndrome | TCM symptom ID | Symptom pinyin name | Modern medical symptoms |
| --- | --- | --- | --- | --- |
| Syndrome 1 | syndrome of liver depression and spleen deficiency | SMTS01179 | Wan Fu Zhang Men | Abdomen Distention |
|  |  | SMTS00240 | Fa Li | Asthenia |
|  |  | SMTS00993 | Shi Shao Fu Zhang | Abdomen Distention |
|  |  | SMTS00573 | Kou Gan | Xerostomia |
|  |  | SMTS00296 | Gan Fu Shui | Ascites |
|  |  | SMTS00405 | Huang Dan | Jaundice |
|  |  | SMTS01249 | Xia Zhi Fu Zhong | Edema Legs, Lower Extremity Edema |
| Syndrome 2 | syndrome of liver-gallbladder dampness heat | SMTS00768 | Pi Fu Huang Ran | Jaundice |
|  |  | SMTS01394 | Xiong Wan Pi Men | Abdomen Distention |
|  |  | SMTS01315 | Xie Lei Zhang Tong | Abdomen Distention |
|  |  | SMTS00707 | Na Dai | Decreased Appetite |
|  |  | SMTS00753 | Ou E | Emesis, Gagging |
|  |  | SMTS00157 | Da Bian Mi Jie | Constipation, Defecation Pain, Colonic Inertia |
|  |  | SMTS00145 | Da Bian Bu Shuang | Defecation Pain |
| Syndrome 3 | syndrome of liver heat and blood stasis | SMTS00573 | Kou Gan | Xerostomia |
|  |  | SMTS00767 | Pi Fu Gan Zao | Eczema Asteatotic, Dry Skin |
| Syndrome 4 | syndrome of spleen deficiency and dampness stagnation | SMTS01178 | Wan Fu Zhang Man | Abdomen Distention |
|  |  | SMTS00954 | Shen Pi | Fatigue, Exhaustion, Lassitude |
|  |  | SMTS00240 | Fa Li | Asthenia |
|  |  | SMTS00707 | Na Dai | Decreased Appetite |
|  |  | SMTS00222 | E Xin | No Vomiting |
| Syndrome 5 | syndrome of liver-kidney yin deficiency | SMTS01945 | Shui Zhong Gu Zhang | Anasarca, Edema |
|  |  | SMTS01354 | Xing Ti Xiao Shou | Emaciation |
|  |  | SMTS00110 | Chuan Cu | Bronchial Asthma |
|  |  | SMTS00573 | Kou Gan | Xerostomia |
|  |  | SMTS00707 | Na Dai | Decreased Appetite |
|  |  | SMTS00905 | She Hong | Glossitis |

**5. Comparison of gene sets associated with HCC disease and its 5 related TCM syndromes with different levels of filtering thresholds.**

To further validate the reliability of the collected syndrome-related genes, we conducted the same calculations using different P-value thresholds, including 0.05, 0.04, 0.03, 0.02, and 0.01. The results consistently demonstrated similar outcomes (**Figure. S3**), emphasizing the reliability of syndrome-related genes identified through SymMap mining, regardless of the stringency of the selection criteria.


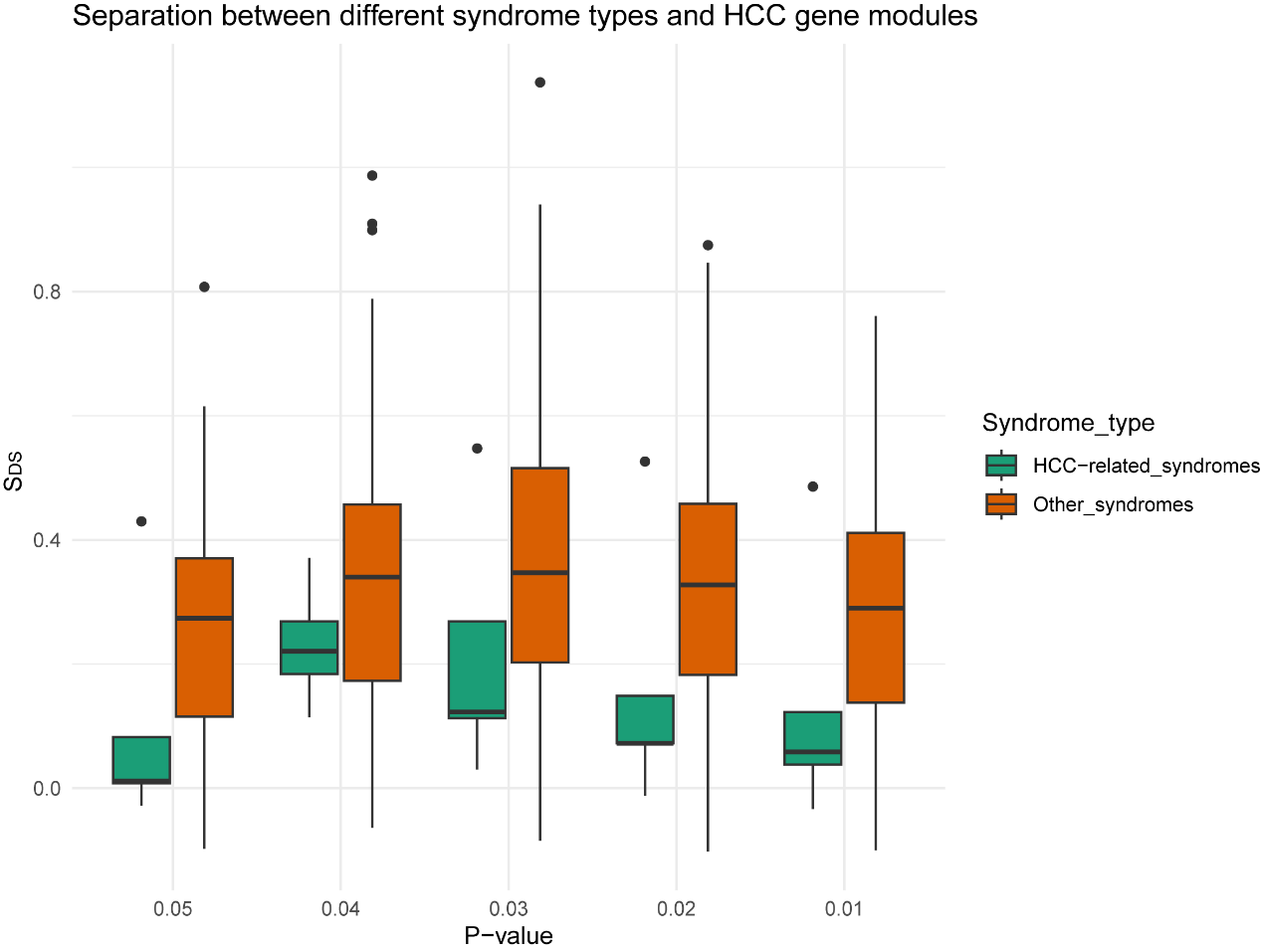


**Figure. S3 The distribution of separation measures, SDS, between HCC genes and those associated with TCM syndromes was examined.** Five sets of boxplots were generated based on five different significance levels (P values), each consisting of two boxes. These two boxes represent two distinct types of comparisons. The first box, depicted in green, assesses the network overlap between HCC genes and those specific to HCC-related TCM syndromes. The second box, in orange, evaluates network overlaps between HCC genes and those associated with 195 other syndromes.

**6. Comparison of formula-HCC topological distances in different databases**

We have expanded our database resources to include HIT 2.0 and the ITCM database. Utilizing consistent inclusion criteria and data collection processes, we have assembled herbs, small molecules, and target data for five TCM formulas from these databases. We then compared the results from these sources with the analysis from the HERB database, focusing on network topology and functional enrichment.

Our comparative analysis revealed that the results from different data sources were largely consistent overall. In terms of topological analysis (**Figure S4**), the targets of the TCM formulas from all databases demonstrated significant proximity to hepatocellular carcinoma (HCC) genes, indicating the potential of TCM formulas to intervene in the complex disease of HCC. This finding is in line with traditional Chinese medicine's holistic approach and its application in treating HCC by targeting multiple pathways simultaneously.


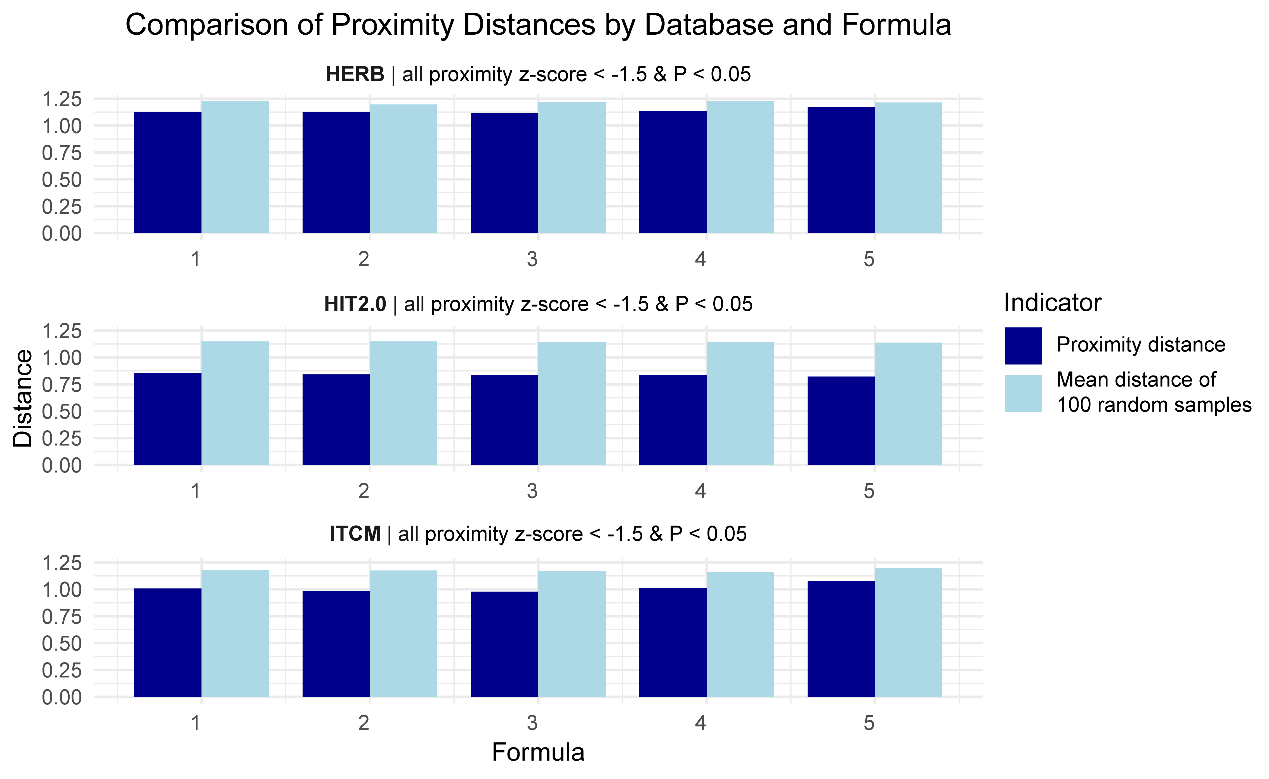


**Figure. S4 Comparison of formula-HCC topological distances in different databases.**

**7. KEGG functional enrichment analysis of disease gene sets and drug target sets**

We performed KEGG functional enrichment analysis on the HCC gene set, the syndrome 1-5 gene sets, the anti-HCC small molecule drug target sets, the formula 1-5 target sets and the overlapping sets of disease-drug respectively (**Figure. S5**). Among them, disease/syndrome-related pathways represent potential dysfunctional pathways in disease states; drug/formulae-related pathways represent potential pathways that drugs can intervene; overlaps-related pathways represent that drug could directly target pathways in related disease states.


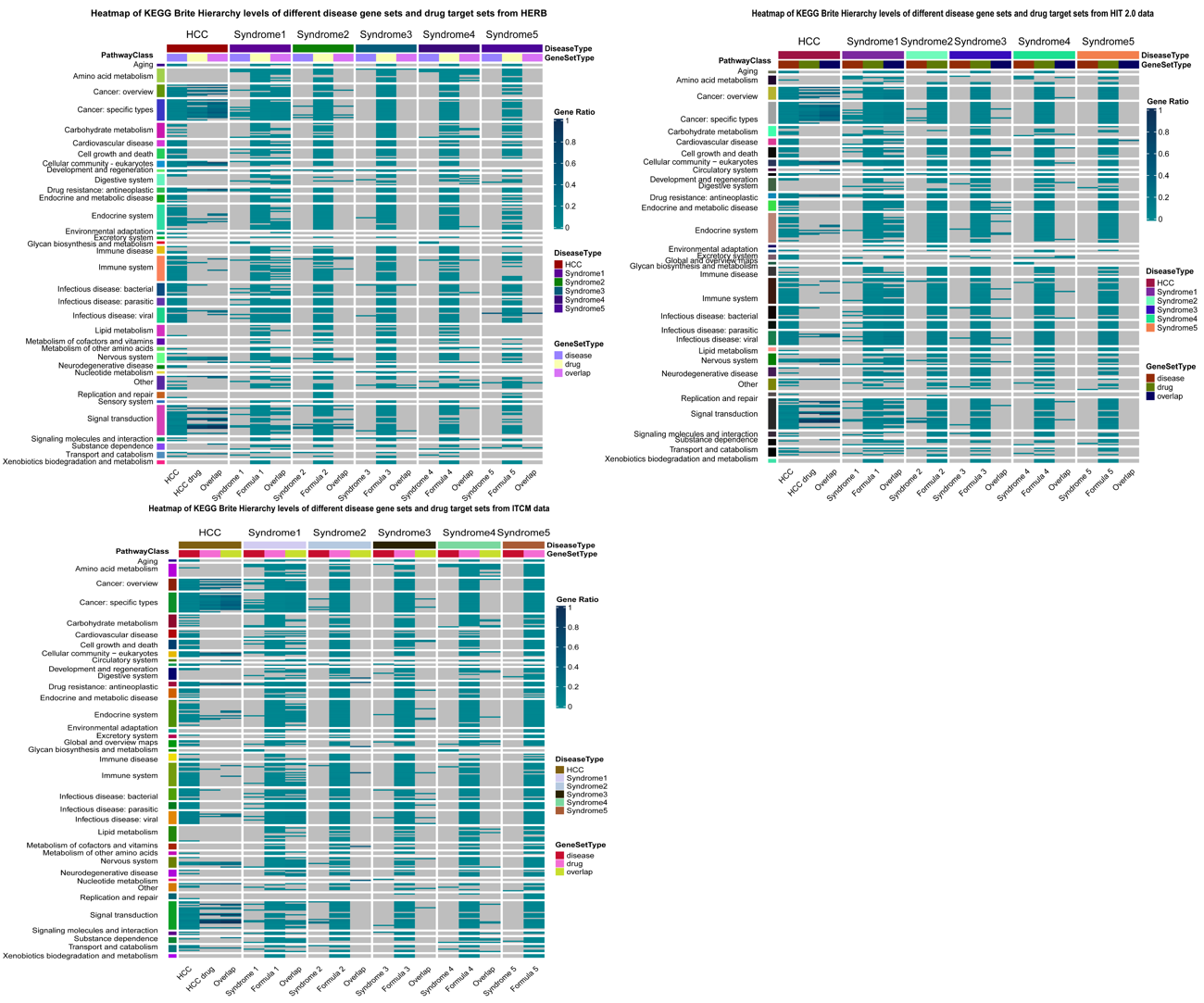


**Figure. S5 Heatmap of drug and disease-associated KEGG pathways.** We compared the functional enrichment analysis clustering results from HERB, HIT 2.0, and ITCM database data.

**8. Experimental literature evidence for the small molecules screened.**

To substantiate the reliability of our results about the small molecules screened, we have conducted an extensive review of the most recent experimental literature, encompassing a variety of in vitro cellular assays and in vivo animal studies (**Table S3**). This comprehensive analysis has provided a wealth of evidence supporting the efficacy of the compounds we have identified.

Given the extensive computational analysis and integration of data from various experimental studies, we are confident that our conclusions are well-supported. The integration of systems biology with traditional Chinese medicine (TCM) principles has yielded insights that are consistent with the known pharmacological effects of the herbal compounds studied. For instance, we have reviewed literature that demonstrates the antitumor effects of compounds such as apigenin, luteolin, nobiletin, and baicalein, which are commonly used in TCM and have been shown to exhibit potent anti-proliferative effects on hepatocellular carcinoma (HCC) cells, both in vitro and in vivo.

We have compiled a table of various experimental evidence that supports the potential of the small molecules we have identified in the fight against HCC. The table includes references to studies that have investigated the effects of these compounds on HCC cells and animal models, providing a foundation for our conclusions:

**Table S3 Experimental evidence of the small molecules anti-HCC**

| PMID | Ingredient | Title | Experimental subject | Study conclusion |
| --- | --- | --- | --- | --- |
| 21861846 | Aloe-emodin | Apoptosis by aloe-emodin is mediated through downregulation of calpain-2 and ubiquitin-protein ligase E3A in human hepatoma Huh-7 cells | Human hepatoma Huh-7 cells | AE exhibited potent anti-proliferative effects on Huh-7 cells via down-regulation of CAPN2 and UBE3A. The findings support the possibility of AE being a chemopreventative agent. |
| 29802937 | Apigenin | Apigenin loaded nanoparticle delayed development of hepatocellular carcinoma in rats | Balb/c mice and Sprague-Dawley rats; HepG2 and Huh-7 | Apigenin loaded nanoparticle delivery substantially controlled the severity of hepatocellular carcinoma and could be a future hope for lingering the survival in hepatic cancer patients. |
| 38394865 | Apigenin and Sorafenib | Apigenin enhances sorafenib anti-tumour efficacy in hepatocellular carcinoma | Not specified in the provided text | Apigenin could enhance the therapeutic efficacy of Sorafenib against liver cancer and may be a promising therapeutic approach for treating HCC. |
| 38823764 | Apigenin | Apigenin inhibits tumor angiogenesis by hindering microvesicle biogenesis via ARHGEF1 | Mouse models of primary liver cancer; Human liver cancer cell lines PLC/PRF/5, Huh7, Hep3B, HepG2 | Apigenin inhibits tumor angiogenesis by hindering microvesicle biogenesis via ARHGEF1, providing a solid foundation for the refinement and practical application of apigenin. |
| 37598926 | β-Sitosterol | β-Sitosterol activates autophagy to inhibit the development of hepatocellular carcinoma by regulating the complement C5a receptor 1/alpha fetoprotein axis | Nude mice xenograft model; HepG2 cells | Our findings demonstrate that β-sitosterol inhibits HCC advancement by activating autophagy through the complement C5a receptor 1/AFP axis. These findings recommend β-sitosterol as a promising therapy for HCC. |
| 38814517 | β-Sitosterol | β-Sitosterol alleviates the malignant phenotype of hepatocellular carcinoma cells via inhibiting GSK3B expression | Huh-7 and HCCLM3 | β-Sitosterol suppressed hepatocellular carcinoma cell proliferation and invasion, and enhanced apoptosis via inhibiting GSK3B expression. |
| 38063438 | β-Sitosterol | β-Sitosterol suppresses hepatocellular carcinoma growth and metastasis via FOXM1-regulated Wnt/β-catenin pathway | Xenograft mouse model; HepG2 cells | β-Sitosterol shows promising potential as a therapeutic candidate for inhibiting HCC growth and metastasis through FOXM1 downregulation and Wnt/β-catenin signalling inhibition. |
| 16242073 | Calycosin | Nanoparticles for the Optical Imaging of Tumor E-selectin | Nude mice; HUVECs; LLC cells | We conclude that ESBP–CLIO (Cy5.5) is a useful probe for imaging E-selectin associated with the LLC tumor, and that E-selectin is expressed not only on endothelial cells but also on LLC cells and human prostate cancer specimens. |
| 31383362 | Luteolin | Regulation of apoptosis and autophagy by luteolin in human hepatocellular cancer Hep3B cells | Hep3B cells, HepG2 cells, NCTC clone 1469 | luteolin-induced ER stress may exert anticancer effects in a p53-independent manner. |
| 30107166 | Luteolin | Luteolin induces caspase-dependent apoptosis via inhibiting the AKT/ osteopontin pathway in human hepatocellular carcinoma SK-Hep-1 cells | SK-Hep-1 cells, AML12 cells | These results demonstrated that luteolin inhibits the AKT/OPN pathway, thereby inducing caspasedependent apoptosis in human HCC SK-Hep-1 cells with little toxicity. |
| 26656468 | Luteolin | Luteolin synergizes the antitumor effects of 5-ﬂuorouracil against human hepatocellular carcinoma cells through apoptosis induction and metabolism | HepG2 and Bel7402 cells | luteolin could synergize the antitumor effects of 5-fluorouracil on HepG2 and Bel7402 cells, which might be related with apoptosis and regulation of 5-fluorouracil metabolism. |
| 23818450 | Nobiletin | Inhibitory Effects of Nobiletin on Hepatocellular Carcinoma In Vitro and In Vivo | SMMC-7721 cells, H22 transplantable tumor in KM mice | Our results suggest that nobiletin has significant inhibitory effects on hepatocellular carcinoma both in vitro and in vivo. |
| 31954513 | Nobiletin | Nobiletin, a novel inhibitor, inhibits HBsAg production and hepatitis B virus replication | HepG2.2.15 cells, HepG2-NTCP cells, mouse model of HBV infection | Nobiletin plays a significant role on HBsAg loss and HBV core DNA clearance, and may work as a novel therapy candidate for the 'functional cure'. |
| 33614661 | Oleic Acid | Anti-tumor Effect of Oleic Acid in Hepatocellular Carcinoma Cell Lines via Autophagy Reduction | Hep3B and Huh7.5, THLE-2 | In conclusion, we report for the first time an autophagy dependent relevant anti-cancer effect of OA in human hepatocellular carcinoma cell lines. |
| 35732625 | Palmitic acid | Proteomic analysis reveals USP7 as a novel regulator of palmitic acid-induced hepatocellular carcinoma cell death | HepG2 | In summary, the present study provides evidence of PA-induced hepatocellular death mediated by deubiquitinase USP7 downregulation and subsequent mitotic catastrophe. |
| 28073184 | Palmitic acid | Functional lipidomics: palmitic acid impairs hepatocellular carcinoma development by modulating membrane fluidity and glucose metabolism | Hep3B, SW480, SW620, AGS, BGC-823, HGC-27, LM3 | Our research support the notion that C16:0 composition is causally connected to HCC growth and metastasis regulation, and open opportunities for diagnosing and treating HCCs by targeting the altered C16:0 metabolism in the near future. |
| 18835982 | Vanillin | Vanillin Inhibits Matrix Metalloproteinase-9 Expression through Down-Regulation of Nuclear Factor-κB Signaling Pathway in Human Hepatocellular Carcinoma Cells | HepG2 cells | In conclusion, vanillin might be a potent antiinvasive agent that suppresses the MMP-9 enzymatic activity via NF-κB signaling pathway. |
| 37759583 | Daidzein | Protective Effect of Daidzein against Diethylnitrosamine/Carbon Tetrachloride-Induced Hepatocellular Carcinoma in Male Rats | Adult male Wistar rats | Due to its antioxidant and anti-inflammatory characteristics, DZ is a promising HCC treatment option for clinical use. |
| 31273958 | Quercetin | Quercetin shows anti-tumor effect in hepatocellular carcinoma LM3 cells by abrogating JAK2/STAT3 signaling pathway | Human hepatocellular carcinoma LM3 cells and nude mice tumor model | Quercetin inhibited hepatocellular carcinoma progression by modulating cell apoptosis, migration, invasion, and autophagy; and its effects were at least partly related with the JAK2/STAT3 signaling pathway. |
| 35204240 | Quercetin | Quercetin Regulates Key Components of the Cellular Microenvironment during Early Hepatocarcinogenesis | Rat model | quercetin has the capability to reduce key components of TME, as well as the expression of ABCC3. |
| 38457248 | Quercetin | Quercetin-induced degradation of RhoC suppresses hepatocellular carcinoma invasion and metastasis | Huh-7, Hep3B, and SK-HEP-1 cell lines; primary HCC cells; patient-derived xenografts (PDXs); nude mice | Our findings provide a rationale supporting quercetin as a potential therapeutic medicine for HCC treatment. |
| 26548344 | Baicalein | Baicalein inhibits hepatocellular carcinoma cells through suppressing the expression of CD24 | HCC cell lines Bel7042 and HepG2 | Our results demonstrate efficient anticancer effects of baicalein on HCC cells and indicate that baicalein suppresses cell growth and cell survival through downregulation of CD24. |
| 36070933 | Baicalein | miR-3,178 contributes to the therapeutic action of baicalein against hepatocellular carcinoma cells via modulating HDAC10 | HCC cells MHCC-97H and SMMC-7721 | Baicalein inhibits cell viability, blocks cell cycle, and induces apoptosis in HCC cells by regulating the miR-3,178/HDAC10 pathway. This finding indicated that baicalein might be promising for treatment of HCC. |
| 31437792 | Baicalein and Baicalin | Baicalein and baicalin promote antitumor immunity by suppressing PD-L1 expression in hepatocellular carcinoma cells | Not specified in the provided content | The study's conclusion is not fully provided in the document content. However, it is mentioned that Baicalein and baicalin decreased STAT3 activity, further downregulated IFN-γ-induced PD-L1 expression and subsequently restored T cell sensitivity to kill tumor cells. |
| 29565220 | Rutin | Implication of nano-antioxidant therapy for treatment of hepatocellular carcinoma using PLGA nanoparticles of rutin | Rat model | Histopathological evaluation indicated reduced incidence of hepatic nodules, necrosis formation, infiltration of inflammatory cells, blood vessel inflammation and cell swelling with RTPLGA-NP treatment along with considerable downregulation in the levels of proinflammatory cytokines. |
| 34512168 | Rutin | Rutin attenuates Sorafenib-induced Chemoresistance and Autophagy in Hepatocellular Carcinoma by regulating BANCR/miRNA-590-5P/OLR1 Axis | Human HCC cell lines | Our findings suggest that rutin could regulate autophagy by regulating BANCR/miRNA-590-5P/OLR1 axis. |
| 32488816 | Rutin | Radiation-synthesis of chitosan/poly (acrylic acid) nanogel for improving the antitumor potential of rutin in hepatocellular carcinoma | Rats | Significant enhanced anti-proliferative, anti-angiogenic, and apoptotic effects were observed for rutin-loaded CAN than free rutin, indicating that this formulation could provide a novel therapeutic approach to serve as a promising agent for treatment of hepatocellular carcinoma. |
| 31561416 | Ursolic Acid | Ursolic Acid Suppresses Cholesterol Biosynthesis and Exerts Anti-Cancer Effects in Hepatocellular Carcinoma Cells | SK-HEP-1, Huh7, Hep3B | UA is a promising cholesterol-lowering nutraceutical for the prevention and treatment of patients with HCC and cholesterol-related chronic diseases. |
| 28755539 | Ursolic Acid and Sorafenib | Simultaneous inhibition of growth and metastasis of hepatocellular carcinoma by co-delivery of ursolic acid and sorafenib using lactobionic acid modified and pH-sensitive chitosan-conjugated mesoporous silica nanocomplex | Murine hepatic cancer H22 cell, human hepatic cancer Huh-7, SMMC-7721, HepG2 cells and human epithelial cancer HeLa cell | Co-delivery of UA and SO by MSN-CS-LA nanocarriers could provide a promising strategy for HCC combinational therapy, especially for the HCC metastasis chemoprevention. |
| 31737119 | Ursolic Acid | Polymeric micelles loading with ursolic acid enhancing anti-tumor effect on hepatocellular carcinoma | Human hepatocellular carcinoma cell line HepG2 and human normal liver cell line L-02 | UA-PMs could markedly inhibit the proliferation and migration of HepG2 cells. In vivo study showed that UA-PMs could significantly inhibit the growth of H22 xenograft and prolong the survival time of tumor-bearing mice, demonstrating great potential in liver cancer therapy. |

**9. Comparison of drug screening methods for different formulae**

We included a comparative analysis of traditional methods with the RWR score to evaluate the performance of different methods in identifying core ingredients.

(1) Traditional Network Pharmacology Computational Methods:

Overlap Analysis: We assessed the overlap between the compounds in the formulas and the genes associated with syndromes to identify potential interactions.

Network Construction: A comprehensive "formula-herb-ingredient-target-syndrome" network was constructed to map out the relationships and interactions.

Topological Parameters: Common topological parameters, including "degree," "betweenness," and "closeness," were calculated to rank the herbal ingredients. The top 5% of herbal ingredients with the highest rankings were considered core ingredients.

(2) Comparative Analysis with RWR Score:

Utilizing the ingredient names and hepatocellular carcinoma (HCC) as keywords, we conducted a literature search on PubMed to identify the number of publications supporting the anti-HCC effects of these ingredients. The count of supporting literature served as a metric to evaluate the drug screening methods.

We created violin plots to illustrate the distribution of the number of publications supporting the anti-HCC effects of the herbal ingredients identified by different methods, as well as the total number of molecules identified (**Figure S6**). The results indicate that the method based on RWR score not only identified a greater variety of herbal ingredients but also those with a significantly higher number of supporting literature, demonstrating its superiority over other traditional network pharmacology parameters.


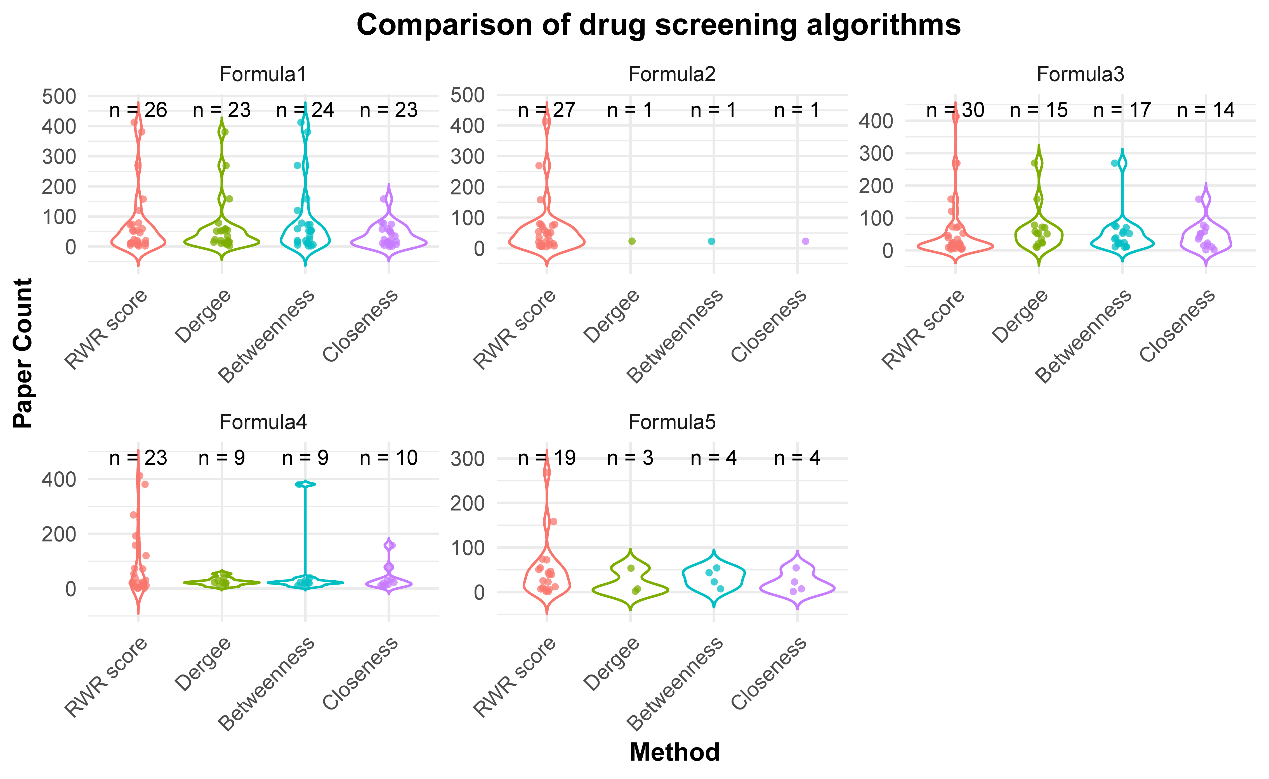


**Figure S6 Comparison of drug screening methods for different formulae**

(3) Rationale and Implications:

The enhanced performance of the RWR score method may be attributed to its ability to integrate both local and global network information, which more accurately reflects the influence of a molecule within the biological context.

The higher number of supporting publications for molecules identified by the RWR score method suggests a stronger alignment with experimental and clinical findings, thereby increasing the confidence in the identified molecules' potential therapeutic effects on HCC.

Our comparative analysis underscores the robustness and reliability of the RWR score method in the context of TCM formula research and its potential application in modern drug discovery.

**10. Structural similarity analysis visualization for herbal ingredients and sorafenib**

In general, similar chemical scaffolds may lead to similar mechanisms of action. We employed a structural similarity analysis visualization method to evaluate this hypothesis.

Using the similarity maps approach outlined by Riniker and Landrum (*Journal of Cheminformatics*, 2013, 5:43), we visualized the atomic contributions to the similarity between nobiletin, baicalein, and sorafenib (Figure S3). This method provides a straightforward and general strategy for the visualization of the atomic origins of fingerprint similarity between molecules.

Our analysis revealed that baicalein exhibits a higher degree of structural similarity to the core scaffold of sorafenib compared to nobiletin, suggesting that they share more molecular fragments in common, which could be the basis for their similar mechanisms of action. The similarity maps indicated that certain regions within the molecules, when removed, would decrease the similarity to the reference compound (sorafenib), highlighting the importance of these regions in maintaining structural and potentially functional similarity.


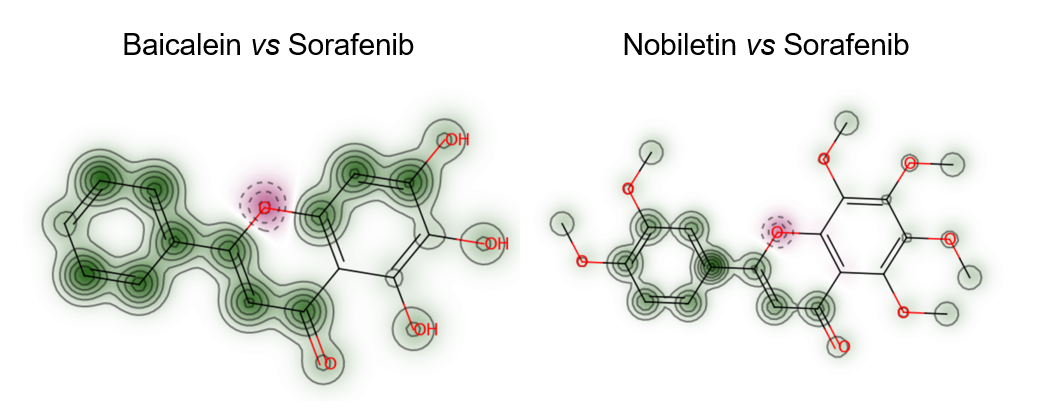


**Figure S7 Similarity maps of active molecules and sorafenib** - Green regions in the similarity maps represent atoms whose removal would decrease the similarity, indicating that these atoms are crucial for maintaining a high level of similarity with sorafenib. This may correspond to key functional groups or structural elements that are important for the activity of these molecules. - Pink regions indicate that the removal of certain atoms would increase the similarity, which could be due to differences at these positions leading to a reduction in overall similarity. These regions may represent structural elements that are less conserved or that contribute differently to the activity compared to sorafenib. - Gray regions suggest that the removal of these atoms has little impact on the similarity, indicating that these parts of the molecule may be more variable and less critical for the activity or the structural similarity to sorafenib.

While nobiletin showed a lower overall similarity to sorafenib, the visualization provided valuable insights into the specific molecular features that contribute to the observed similarity, which could guide the discovery of new structural scaffolds with potential therapeutic relevance.

**Legends for Supplementary Data:**

Supplementary Data 1: Gene sets of diseases and TCM syndromes.

Supplementary Data 2: Disease-drug pairs.

Supplementary Data 3: Target sets of small molecule drugs, TCM formulae and herbs from HERB.

Supplementary Data 4: Protein-protein interactions.

Supplementary Data 5: Target sets of TCM formulae and herbs from HIT 2.0 and ITCM.
